# Protein tyrosine kinase inhibitor resistance in malignant tumors: molecular mechanisms and future perspective

**DOI:** 10.1038/s41392-022-01168-8

**Published:** 2022-09-17

**Authors:** Yang Yang, Shuo Li, Yujiao Wang, Yi Zhao, Qiu Li

**Affiliations:** 1 Cancer Center, West China Hospital, Sichuan University, No. 37, GuoXue Xiang, Chengdu, Sichuan China; 2 West China Biomedical Big Data Center, Sichuan University, No. 37, GuoXue Xiang, Chengdu, Sichuan China; 3 Second Clinical Medicine College, Lanzhou University, Lanzhou, Gansu China; 4 Nephrology, West China Hospital, Sichuan University, No. 37, GuoXue Xiang, Chengdu, Sichuan China; 5 First Clinical Medicine College, Lanzhou University, Lanzhou, Gansu China

**Keywords:** Cancer, Cancer

## Abstract

Protein tyrosine kinases (PTKs) are a class of proteins with tyrosine kinase activity that phosphorylate tyrosine residues of critical molecules in signaling pathways. Their basal function is essential for maintaining normal cell growth and differentiation. However, aberrant activation of PTKs caused by various factors can deviate cell function from the expected trajectory to an abnormal growth state, leading to carcinogenesis. Inhibiting the aberrant PTK function could inhibit tumor growth. Therefore, tyrosine kinase inhibitors (TKIs), target-specific inhibitors of PTKs, have been used in treating malignant tumors and play a significant role in targeted therapy of cancer. Currently, drug resistance is the main reason for limiting TKIs efficacy of cancer. The increasing studies indicated that tumor microenvironment, cell death resistance, tumor metabolism, epigenetic modification and abnormal metabolism of TKIs were deeply involved in tumor development and TKI resistance, besides the abnormal activation of PTK-related signaling pathways involved in gene mutations. Accordingly, it is of great significance to study the underlying mechanisms of TKIs resistance and find solutions to reverse TKIs resistance for improving TKIs efficacy of cancer. Herein, we reviewed the drug resistance mechanisms of TKIs and the potential approaches to overcome TKI resistance, aiming to provide a theoretical basis for improving the efficacy of TKIs.

## Introduction

Malignant tumors are the second leading cause of death and a public health concern worldwide. Anti-tumor therapy is the core means of reducing tumor mortality, and related research mainly focuses on molecular level^1^. Oncogenic mutations of cell signaling molecules lead to abnormal clonal proliferation, as the main characteristic of tumor cells. Some signaling proteins and pathways are susceptible to oncogenic mutations in normal cells, particularly molecules that control cell growth, differentiation, and developmental signals, one of which is protein tyrosine kinases (PTKs).^1^ PTKs are a class of proteins with tyrosine kinase activity whose function is tightly regulated in normal cells. Perturbation of PTKs function by mutations and other genetic alterations can lead to malignant transformation^1,2^. Previous studies have shown that more than 80% of oncogenes and proto-oncogenes finally increase PTKs expression.^2^ Therefore, inhibition of PTK overactivity is a major strategy for treating cancer.

Tyrosine kinase inhibitors (TKIs) are target-specific inhibitors of abnormal PTKs. As homologs of adenosine triphosphate (ATP), TKIs competitively occupy the ATPs-binding site of PTKs and block PTK-mediated signaling pathways in cancer cells, thereby inhibiting their growth and proliferation. TKIs have significant advantages over traditional chemotherapeutic agents, including high efficiency, low toxicity, and high specificity. Presently, TKIs are widely used for treating leukemia, non-small-cell lung cancer (NSCLC), renal cell carcinoma (RCC), gastrointestinal stromal tumor (GIST), breast cancer, and hepatocellular carcinoma (HCC), and their clinical application is rapidly growing.^3–5^

Tumor cells gradually develop resistance during TKIs therapy; however, the time frame for developing acquired drug resistance is not long. For instance, the effective time frame for treating NSCLC with first-generation epidermal growth factor receptor (EGFR) TKIs does not exceed one year.^6^ In addition, a small proportion of tumor cells are primarily resistant to TKIs. NSCLCs with Bcl-2-interacting mediator of cell death (BIM) deletion polymorphisms are primarily resistant to osimertinib.^7^ TKI resistance is a leading cause of recurrence, progression, treatment failure, low compliance and mortality among cancer patients. The mechanisms of TKIs resistance are complex. Previous studies have affirmed the relationship between TKI resistance and target-gene mutations. A growing body of evidence suggests that other factors, such as tumor microenvironment (TME) and epigenetics, are also involved in TKI resistance.^8^

Despite the remarkable effectiveness of TKIs in targeted therapy, TKIs resistance is increasingly growing. Here, we summarize the underlying mechanisms of TKIs resistance and discuss the potential approaches to overcome it. We aim to provide a theoretical basis for future research and management of TKIs resistance.

## PTK, TKI, and cancer

### PTK and cancer

**Fig. 1:**
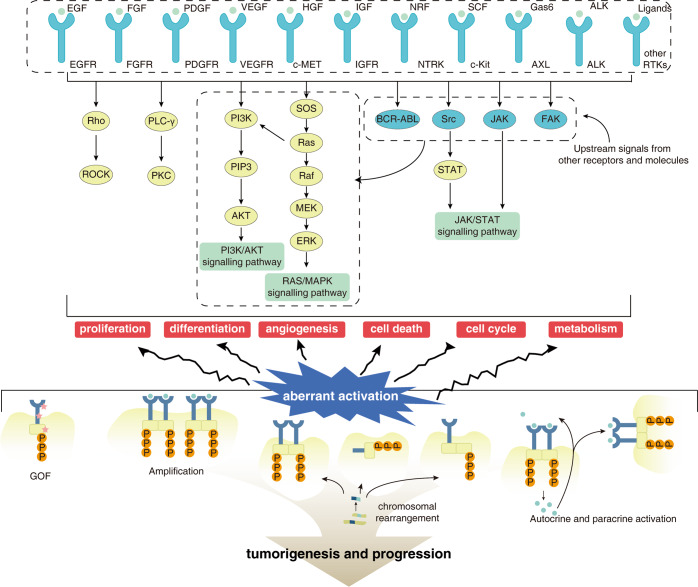
The relationship between PTK and tumors. The normal activation and inactivation of PTK is essential to maintain normal cellular function. PTK activation mutations include gain-of-function mutations, genomic amplification and overexpression, chromosomal rearrangements (gene fusions), and ligand autocrine/paracrine loops

PTK activity is critical for the proper function of signal transduction pathways associated with cell proliferation, differentiation, and survival.^9^ Based on PTK location in the cell, PTKs are classified into receptor tyrosine kinases (RTKs) and non-receptor tyrosine kinases (NRTKs). Located in the cell membrane, RTKs are divided into subfamilies based on the molecular characteristics of their extracellular structural domains. Previous studies classified 58 known human RTKs into 20 subfamilies.^10^ However, based on an in-depth functional analysis, three RTKs of the lemur tail kinase (LMTK) family were found to phosphorylate serine/threonine residues instead of tyrosine residues, resulting in the classification of 55 RTKs into 19 subfamilies.^11^ In addition to RTKs, there are a large number of NRTKs in cells, including ABL Proto-Oncogene (Abl), FES Proto-Oncogene (Fes), Janus Kinase (JAK), Focal adhesion kinase (Fak), and SRC Proto-oncogene (Src).^9^ NRTKs regulate various cellular functions, including cell proliferation, differentiation, adhesion, migration, and apoptosis by coupling with RTKs or other membrane receptor proteins such as G protein-coupled receptors. Moreover, NRTKs are also involved in T- and B-cell activation signaling pathways, thereby modulating immune response (Fig. 1).^9,12^

Some PTKs mutations can keep PTKs in a persistently “active” state, causing overactivation of downstream pathways, and everlasting proliferation, differentiation, angiogenesis, and tumorigenesis.^1^ PTKs overactivation is mediated by four major types of mutations:^13^ (I) Gain-of-function (GOF) mutations include anaplastic lymphoma receptor tyrosine kinase (ALK), R1275Q, and F1147L mutations in neuroblastoma (NB).^14^ Some GOF mutations can increase the sensitivity of TKI and signaling transduction. For instance, deletion of four highly conserved amino acids, LREA, in EGFR exon 19 and the L858R point mutation in exon 21 of lung cancer improves therapeutic response to EGFR-TKIs.^15,16^ (II) Genomic amplification and overexpression is the second major type of mutation. For example, overexpression of EGFR and vascular endothelial growth factor receptor 2 (VEGFR2) is associated with medullary thyroid carcinoma.^17^ (III) Chromosomal rearrangements (gene fusions). The Philadelphia chromosome (BCR-ABL fusion) is a well-known example of gene fusion causing chronic myeloid leukemia (CML). MASC (Mammary analog secretory carcinoma) has an ETS variant transcription factor 6 (ETV6)-neurotrophic receptor tyrosine kinase 3 (NTRK3) gene translocation.^18^ In addition, partial duplications within genes are also a type of chromosomal rearrangement, and kinase domain duplication (KDD) of RTKs may be a novel mechanism for constitutive activation of RTKs.^19,20^ In a genomic analysis of 114,200 solid tumors, KDD was found in 0.62% of tumors, particularly brain tumors, predominantly in EGFR, platelet-derived growth factor receptor alpha (PDGFRA), and fibroblast growth factor receptor 3 (FGFR3).^21^ (IV) Ligand autocrine/paracrine loop. For example, fibroblasts can release hepatocyte growth factor (HGF), which binds to MET proto-oncogene (MET) in cancer cells and provokes a continuous survival benefits.

Protein tyrosine phosphatases (PTPs) are the physiological antagonists of PTKs. PTPs are responsible for attenuating the effects of PTKs by dephosphorylating target proteins. The subtle antagonistic function of PTKs and PTPs strictly regulates the phosphorylation level of intracellular proteins and is pivotal for maintaining normal signaling. However, antagonistic function of PTPs cannot completely attenuate PTKs overactivity caused by the above mechanisms.^13^ As endogenous PTPs cannot inhibit the sustained activation of PTKs, exogenous PTK inhibition is necessary.

### TKI and cancer

The kinase structural domains of PTKs are responsible for transferring the phosphate group of ATP to the tyrosine residue of downstream signaling molecules. Regarding the structural similarity of TKIs and ATPs, TKIs can competitively bind to the kinase structural domains to prevent downstream pathway activation and inhibit tumor growth. Kinase inhibitors, including TKIs, are often classified into six types based on the mechanism of action, but there is no uniformity.^22–24^ Type I kinase inhibitors, including cabozantinib, crizotinib, and gefitinib, compete with substrates and bind to the ATP-binding pocket of the active conformation. Type II kinase inhibitors such as imatinib, sorafenib, and nilotinib, bind to the inactive conformation of protein kinases. The binding sites of types III and IV are not in the ATP pocket and function through allosteric mechanisms. However, due to the complexity of the allosteric mechanisms, there are few approved TKIs except for asciminib approved for CML.^25^ Types IV and V kinase inhibitors can form covalent bonds with kinase sites, thus irreversibly altering target activity. These kinase inhibitors such as osimertinib, afatinib, ibrutinib, and acalabrutinib possess better pharmacokinetic properties than reversible inhibitors.

TKIs bind to abnormal PTKs to inhibit downstream pathways. The PI3K/AKT and RAS/MAPK signaling pathways are two major PTKs-related pathways that can induce proliferation, inhibit apoptosis, promote angiogenesis, and regulate various cellular functions. Consistently, TKIs can reverse these alterations. Additionally, some TKIs can also modulate the immunosuppressive microenvironment of tumors,^26,27^ alter the molecules expression level of immune cell surface through the TME and promote the antitumor immune response. Interestingly, a clinical trial (NCT0301333) demonstrated that TKIs and antibiotics influenced the therapeutic response of RCC to immunotherapy by affecting the composition of the gut microbiota.^28^ A recent study in glioblastoma (GBM) cell lines provided new insights into the function of TKIs, other than the PTKs inhibitory role. The study revealed that some TKIs can activate general control nonderepressible 2 (GCN2), which activated integrated stress response (ISR), resulting in cell death.^29^

Although different TKIs have similar mechanisms of action, they differ in terms of targeting kinase profiles, pharmacokinetics (PK), and side effects.^30^ For example, osimertinib is a third-generation TKI targeting EGFR for NSCLC with specific mutations. Imatinib is highly selective for BCR-ABL mutation and is the best choice for CML. However, multiple PTK abnormalities exist in some tumors, and single-target therapy is sometimes inadequate. Multi-target TKIs that cover a broader range of PTK abnormalities can provide more potent inhibitory effects and have a broader range of indications. For instance, sorafenib can target various RTKs, including VEGFR and PDGFR, thereby inhibiting cancer cell proliferation and angiogenesis. Sorafenib is effective in HCC, RCC, and thyroid cancer (TC).^31^ As of 2021, 76 TKIs have been administered for treating various types of cancer, and more are in the phase of clinical trials.^32^ Entering the human body with a clear target, the small molecule TKIs have only limited nonspecific toxicity. The favorable safety allows TKIs to be easily combined with other treatment modalities, such as chemotherapy, immunotherapy, and radiotherapy. TKIs significantly prolong survival and improve the quality of life among cancer patients.

Although TKIs are highly effective in cancer, some issues such as adverse events and tumor resistance to TKIs still require the vigilance of clinicians. The most common adverse events of TKIs are dermatologic complications (such as folliculitis, paronychia, and periorbital edema) and hematologic side effects (such as neutropenia, gastrointestinal symptoms, and hypothyroidism).^30^ Management of TKI-related adverse events is of clinical significance for cancer patients. In addition, some patients weakly respond to TKIs or gradually become insensitive to TKIs as the manifestations of TKIs resistance.^33,34^ TKIs resistance leads to recurrence or rapid tumor progression, which seriously affects the survival of patients. Therefore, finding solutions for TKI resistance is an urgent priority.

## Mechanisms of TKI resistance

**Fig. 2:**
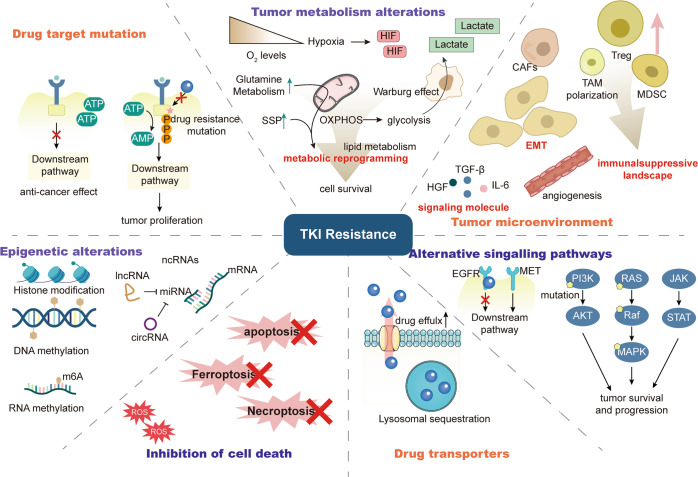
Overview of the mechanisms of tumor resistance to TKIs. The mechanism of tumor resistance to TKIs is complex. The most direct mechanism is the mutation of TKI drug targets, which will lead to the inability of TKIs to bind and function, causing drug resistance. Tumors can also cause resistance by reducing intracellular TKI concentrations, such as enhanced drug efflux and lysosomal isolation. There are also other factors outside the tumor cell that can affect the tumor response to TKI. The tumor microenvironment is important for TKI resistance, with crosstalk of various cellular components within the TME, which on the one hand can complement the growth signal of cancer cells, and on the other hand, the immunosuppression caused by the TME prevents immune cells from killing tumors. In addition, tumor cells within the TME can undergo a series of adaptive changes, such as metabolic reprogramming and EMT, to resist cell death. Tumor cells can also activate downstream pathways through signaling bypass or without relying on PTK, leading to drug resistance. Finally, epigenetics permeates almost all drug resistance mechanisms through unique regulatory mechanisms

TKIs resistance mechanisms are highly complex, including abnormal drug metabolism, cell death, epigenetics, etc. Multiple factors lead to resistance to TKIs (Fig. 2). The mechanisms of drug resistance extensively vary among individuals. Identification of drug resistance mechanisms can improve the clinical efficacy of TKIs. Here, we elaborately review the current mechanisms of TKIs resistance.

### Reduced PTK-binding capacity of TKI

TKIs can generally enter the cell and bind to abnormally activated PTKs. However, extensive mutations in some PTKs decrease TKI binding capacity as a direct mechanism of TKI resistance (Table 1). On the one hand, intrinsic resistance occurs due to changes in kinase conformation and activity caused by specific mutations in PTK. For example, EGFR exon 20 insertion is observed in 3% of patients with lung adenocarcinoma and 9% of patients with EGFR mutations, leading to third-generation EGFR-TKI resistance.^35^ Deletions of exon 16 of human epidermal growth factor receptor 2 (HER2) splice variant (Δ16HER2) lead to primary resistance to lapatinib in breast cancer cell lines. It is also associated with acquired resistance to Src-TKI, saracatinib.^36^ In addition, KIT Proto-Oncogene (KIT) exon 9 mutation and PDGFRA D842V substitution are associated with intrinsic resistance to imatinib in GIST.^37^ Similarly, ALK L1196 M/G1202R causes primary resistance of NSCLC to lorlatinib.^38^ PDGFRB C843G can also lead to Ph-like acute lymphoblastic leukemia (ALL) resistance to all ABL TKIs, which is a classic example of this mechanism.^39^

**Table 1:** On-target gene mutations of TKI resistance

RTKs		Other mutations before gatekeeper	Gatekeeper mutations	Other mutations after gatekeeper	solvent-front mutations	covalent binding site mutations	Gene fusion mutation
EGFR		L692V, E709A/K, L718Q/V, L719A, S768,	T790M	G796S, L792X/F/H/R/Y/V/P, G796R/S	G196S/R	C797S/X	–
FGFR	FGFR1	N546K/H	V561F/M		—	—	—
	FGFR2	M536I, M538I, K462, I548V, N549K/H, N550H	V564F/I	E565A, I567, N568, V581, E584G, S587, L617V, L618M, V652L, K659M, K660E/M, K678M, H682L, K714R, E719G	—	—	—
	FGFR3	—	V355M	V555M	—	—	—
	FGFR4	—	V550L/E/M	C552	—	—	—
ALK		L1122, 1146 K, L1152V/Q, C1156Y, I1171T/N, L1174V, V1180	L1196M	L1198F, F1245V, G1269A/S	G1202R, D1203N	—	EML4-ALK
ROS1		L1174F, L1951, S1986F/Y, L1986F/L, F2004C/I/V	L2026M, G2026M	L2086F, L2155S	G2032R/K, D2033N	—	—
RET		E732K, V738A	V804L/M	Y806N, G807V, G810S, V871, M918T, S904F, F998V	L730V/I, G810A/C/S/R,	—	—
NTRK	NTRK1	V573, G595R	F589L	G667C/S, A608D	G595R/A	—	
	NTRK2	V601	F633L	G709C	G639R	—	
	NTRK3	—	F617L	G696A	G623R	—	
BTK		Less frequently	—	—	—	C481S	—
BCR-ABL		M244, L248, G250, Q252, Y253, E255, V299	T315I	F317, A337, M351, M355, F359, H396, W464, P465, V468, I502	—	—	—
FLT		D200N, K429F, Y572C, L601F,	F691I/L	D835Y/V, Y842C/H	—	—	—
KIT		V653A, V654A, T669I	T670I	D816H, D820A, N822K, Y823D, A829P	—	—	—

*EGFR* epidermal growth factor receptor, *FGFR* fibroblast growth factor receptor, *ALK* anaplastic lymphoma receptor tyrosine kinase, *ROS1* ROS proto-oncogene 1, *RET* RET proto-oncogene, *NTRK* neurotrophic receptor tyrosine kinase 1, *BTK* bruton tyrosine kinase, *FLT* Fms related receptor tyrosine kinase 1, *KIT* KIT proto-oncogene, *EML4* EMAP like 4.

PTK can develop secondary mutations during treatment with TKI, leading to acquired drug resistance. The gatekeeper mutation is one of the first identified acquired resistance mutations of TKI targeted kinase.^40^ The most typical gatekeeper mutation is substituting a smaller amino acid with an amino acid with a larger hydrophobic residue. This mutation results in a spatial blockade that hinders the formation of a hydrogen bond between PTK and TKI.^41^ EGFR T790M mutation in exon20 causes spatial blockade to the first- and second-generation EGFR TKIs. In the coincidence of activating mutations like L858R, T790M lowers Km _[ATP],_ the ATP concentration to achieve a half-maximal reaction rate. Consequently, the affinity of ATP increases and the efficacy of TKI decreases.^42,43^ FGFR V564F, KIT T670I, and Ret Proto-Oncogene (RET) V804M are also gatekeeper mutations,^44,45^ but they are not as common as T790M and still lack of enough studies.

Solvent-front mutation can be observed in RET. The solvent-front refers to the kinase residues exposed to the solvent or the region of the kinase surface to which TKIs bind. L730 lies at the top of the solvent-front of RET and binds to nintedanib by hydrophobic interactions with piperazine, phenylamino, and phenyl groups.^46^ Thus, the RET L730V mutation results in nintedanib resistance. Another highly mutated site of RET solvent-front is at G810. The G810A mutation has an additional methyl group, which can form new hydrophobic bonds with the methyl and phenyl rings in nintedanib. Despite vandetanib, nintedanib has these methyl and phenyl rings, so G810 only causes resistance to vandetanib but no effects to nintedanib. Substitution of G810 by a more polar amino acid like serine prevents its TKI binding capacity and improves resistance to vandetanib and nintedanib.^46^ Moreover, selpercatinib and pralsetinib are specific RET TKIs that were recently approved. G810S/C/R mutation causes selpercatinib resistance, and L730 V/I mutation leads to pralsetinib resistance.^47,48^ In addition to RET, solvent-front mutations also exist in EGFR, ALK, ROS1, and NTRK and contribute to drug resistance, such as EGFR G796S/R, ALK G1202R, NTRK1-G595R, NTRK2-G639R, NTRK3-G623R, ROS1 G2032R, and ROS1 D2033N.^49,50^

Except for classic mutations of the kinase structural domain, some newly identified mutations can also weaken drug-target interactions by a spatial blockade. L792X/F, G796S, L718Q, and S768I, are some of the acquired mutations of EGFR and E565A, L617V, K641R, and K659M are the structural domain mutations of FGFR2 kinase.^35,51,52^ ALK resistance mutations include point mutations such as G1269A, I1171T/N, L1196M, C1156Y, F1245V, and F1174V, and compound mutations like L1196M/G1202R, and L1152V/Q1146K.^53,54^ As for ROS1, G2032R, S1896F, and L2000V lead to lorlatinib resistance,^55,56^ G2032R, L2086F, G2026M, D2033N, S1986F/Y, and L1174F cause crizotinib resistance.^57^ L2086F can cause resistance to lorlatinib, crizotinib, and entrectinib but not cabozantinib.^56^ F2004C/I/V contributes to the resistance of entrectinib, crizotinib, and cabozantinib.^58^ Among RET kinase mutations, L730I, V738A, V804 L/M, Y806N, and G810S mutations are pan-resistant to cabozantinib, lenvatinib, vandetanib, and nintedanib. Additionally, l730V, E732K, A807V, G810A, V871I, M918T, and F998V mutations cause resistance to one or some of these drugs. Interestingly, the V871I, M918T, and F998V mutations are far from the ATP-binding pocket but can also inhibit TKI binding.^59^ In addition, NSCLC patients with positive ALK carrying the EML4-ALK fusion gene can rapidly develop alectinib resistance within three months.^60^ The KIT V654A and D820A mutations can cause GIST resistance to imatinib.^61,62^ The NTRK G623R mutation can lead to MASC resistance to entrectinib, but the next-generation NTRK-TKI selitrectinib is still effective in MASC with this mutation.^18,63^

Mutation in the drug target site still decreases the efficacy of irreversible inhibitors. Osimertinib, a third-generation EGFR covalent inhibitor, binds to the C797 residue in the ATP-binding site of EGFR and potently inhibits receptor activation, which can be applied to positive T790M EGFR. The missense mutation in exon 20 C797S prevents covalent bond formation with EGFR and leads to osimertinib resistance.^35^ Bruton tyrosine kinase (BTK) TKIs like ibrutinib, acalabrutinib, and zanubrutinib, constitute another class of covalent inhibitors. They irreversibly and covalently bind to the C481 site of BTK. Therefore, drug resistance can occur in the presence of C481 mutation.^64^

The resistance mechanisms are not limited to spatial interference. RET S904F mutation increases the affinity of RET to ATP and leads to RET-TKI vandetanib resistance.^65^ FGFR2 N550H attenuates the auto-inhibitory mechanism of the kinase hinge region and allows the kinase to activate, preventing dovitinib from binding its target.^66,67^ Studying the role of ABL mutations in imatinib resistance showed that individual resistance mutations do not significantly affect drug binding capacity. Alterations in ABL kinase activity and the affinity for ATP and its kinase substrates can be observed together. The coincidence of these alterations changes the energy landscape of the kinase, which can be observed in imatinib resistance.^68^ This “resistance accumulation” mechanism may apply to a broader scope.

There are four possibly effective countermeasures to resistance mutations. For instance, the standard treatment is platinum-based chemotherapy in patients with T790M-negative tumors at the time of acquired resistance. For T790M mutation, irreversible inhibitors are still effective, and osimertinib is recommended by several countries based on clinical trials.^43^ Although C797S secondary mutation decreases the efficacy of osimertinib, recent findings from clinical studies show that a combination of first and third-generation EGFR TKI is an effective option if the T790M and T797S mutation *loci* are on different chromosomes, known as *trans* mutation. But EGFR with T790M and T797S mutations on the same chromosome, known as *cis* mutation, is insensitive to all EGFR-TKIs. In addition, in GIST with KIT T670I, cabozantinib can overcome imatinib resistance.^69^ As previously discussed, some resistance mutations may cause resistance to only one TKI and have little effect on other TKIs. In this case, changing to a more sensitive TKI may be effective. Second, TKI combined with other treatments will be discussed in the following sections. Third, switching to other treatments by identifying other potentially targetable mechanisms of resistance or participating in clinical trials might be suitable choice.

Finally, developing new TKIs or finding new sites of action is a promising solution for all mutation-related resistance. A Phase I clinical trial showed an unprecedented and durable clinical benefit when treating PDGFRA D842V-mutant GIST with avapritinib.^70^ The mice model showed that the next-generation ROS/ALK inhibitor SAF-189S had comparable efficacy to lorlatinib against crizotinib-resistant lung cancer with ROS G2032R-mutation.^71^ The next-generation ALK-TKI XMU-MP-5 led to marked cancer regression in mice with EML-ALK mutations.^72^ Ripretinib broadly inhibited activating mutations in KIT and PDGFRA and the efficacy was shown in preclinical studies.^73^ LY2874455 is a newly discovered pan-FGFR inhibitor overcoming FGFR gatekeeper mutations.^74^ Moreover, fourth-generation EGFR-TKIs are assumed to target PTKs with drug-resistance mutation by allosteric mechanisms. Some of them, such as DDC-01-1163, JBJ-04-125-02, and 4-(1-ethylsulfonyl-3-indolyl)-2-phenylaminopyrimidines are currently used in preclinical studies.^75–77^ Fourth-generation EGFR-TKIs combined with other drugs are promising, but more clinical trials are needed to consolidate the effectiveness.

In addition, most of the previously developed TKIs have tumor-specific indications, but new-generation TKIs have mutation-specific indications. For example, larotrectinib can effectively treat all solid tumors carrying NTRK fusion genes,^78–81^ and repotrectinib (TPX-0005) is a next-generation broad-spectrum inhibitor of ALK/ROS/NTRK that can treat a variety of solid tumors carrying ALK, ROS1, and NTRK mutations.^50^ TPX-0046 is a next-generation RET/SRC TKI with a three-dimensional macrocyclic structure distinct from selpercatinib and pralsetinib. It has potent activity against multiple RET mutations. Repotrectinib is also a next-generation multitarget inhibitor that overcomes resistance in ALK/ROS/NTRK-positive solid tumors with solvent-front mutations.^50^ However, repotrectinib can overcome gatekeeper and solvent-front mutations, it remains insensitive to NTRK1 G667C and NTRK3 G696C in DGF motifs.^82^

In general, resistance mutations may overlap and vary between TKIs, which may be related to the types of the kinase. Therefore, if a resistance mutation occurs during TKI therapy, changing the TKI may be helpful. However, it is not a long-term solution. Cancer cells gradually develop resistance mutations even with new-generation TKIs. Moreover, PTK resistance mutations can be associated with abnormal activation of non-target PTKs. Herein, EGFR mutation accompanied by MET amplification attenuates the efficacy of a single TKI to inhibit the proliferation caused by abnormal activation of other PTKs. A combination of other drugs, such as MET-TKIs, can partly overcome the resistance in such cases. Because of irreversible resistance mutations, proper pre-drug resistance administration and regular monitoring of drug-resistant mutations are pivotal to circumventing drug resistance. Studies on different EGFR-TKIs and pazopanib indicated that compared to continuous dosing, a higher dose of TKI or intermittent pulse known as “drug holidays” can increase the efficacy of targeted therapy, delay treatment resistance, and reduce toxicity.^83–85^

Early monitoring of drug resistance mutations can determine the best treatment regimen, particularly with the development of high-quality detection methods in recent years. Earlier, the biopsy was an effective method to detect mutations. However, tissue samples are not often enough to systematically represent the mutational status of the entire tumor, and the sample amount is also insufficient for molecular detection. In contrast, cell-free DNA (cf-DNA) and next-generation sequencing (NGS) are now available to detect circulating tumor DNA (ctDNA) for comprehensive detection of molecular alterations.^43,86^ Several studies support the potential of ctDNA-NGS for rapid selection of new drugs after TKI treatment failure and predicting the clinical outcomes.^87,88^ In addition, the Zebrafish patient-derived xenograft (ZTX) platform, which is based on patient-derived xenograft (PDX), is a high-efficacy preclinical model for studying resistance mechanisms for targeted therapies. It estimated the erlotinib treatment outcome and tumor invasion in patients with lung cancer within three days, and found it had high sensitivity (91%) and specificity (62%).^89^ It also effectively predicted individualized tumor regression.^90^ More importantly, we should understand when TKIs must be used to treat tumors with RTK mutations. There is heterogeneity in the initial response to TKIs for different types of mutation.^91^ Taking EGFR as an example, patients with typical L858R and EX19del mutations sufficiently respond to TKIs, while patients with some atypical EGFR mutations inadequately respond to these TKIs. For atypical PTK mutations, some existing TKIs may not be suitable. It is impossible to predict how much a patient will benefit from TKI therapy before TKIs are administered. However, the locus of the mutation can help to predict treatment outcomes. Accordingly, it is necessary to establish a classification system of PTK mutation to predict TKI sensitivity.^91^

### Abnormal activation of PTK-related signaling pathways

The function of TKIs is to inhibit the activation of downstream pathways caused by abnormally activating PTKs. The PI3K/AKT, RAS/MAPK/ERK, and JAK/STAT signaling pathways are the most important pathways. However, PTK inhibited by TKI cannot suppress a signaling pathway activated by downstream molecule mutation. Therefore, RTK-independent overactivation of the downstream pathway is unresponsive to TKI. Sometimes several PTKs activate the same downstream pathway, and the inability of a TKI to inhibit all those PTKs leads to persistent activation of the pathway and drug resistance. On the other hand, activation of PTK-related downstream pathways by other signaling pathways can bypass the TKI and cause drug resistance. Downstream signaling pathway activation by other PTKs or other signaling pathways is named bypass resistance. Furthermore, many proteins can move into the nucleus in response to signals that regulate gene expression. Nuclear translocation, as part of the signaling pathway, has also been associated with TKI resistance. Here, we have summarized several types of aberrant activation of PTK-related signaling pathways which can be resist to TKIs.

#### Abnormal RTK-independent activation of downstream pathways

PI3K/AKT and RAS/MAPK pathways can be activated without upstream stimulation when the downstream signaling molecules are abnormally activated. In addition, each pathway has its specific negative regulators. Without the effective function of these negative regulators, TKIs-mediated inhibition of upstream molecules will be less effective. These two states are defined as abnormal RTK-independent activation of downstream pathways.

Mutations of downstream members of the pathway can activate the pathway, independent of the upstream molecules. Phosphatidylinositol-4,5-bisphosphate 3-kinase catalytic subunit alpha (PIK3CA) encodes the catalytic subunit of PI3K. Mutations of PIK3CA were found in EGFR-TKI-resistant NSCLC cell lines, MET-TKI capmatinib-resistant NSCLC cell lines, regorafenib-resistant CRC cell lines, KIT/PDGFR-TKI-resistant GIST cell lines, imatinib-resistant Ph^+^ ALL cell lines, and HER-TKI neratinib-resistant HER2^+^ breast cancer cell lines,^92–97^ resulted in enhancing PI3K kinase activity. Mechanistic target of rapamycin kinase (mTOR) L1433S mutation activates AKT and leads to EGFR-independent osimertinib resistance.^98^ Furthermore, the serum- and glucocorticoid-regulated kinase 1 (SGK1) is functionally and structurally similar with AKT. Despite crenolanib-mediated inhibition of AKT, breast cancer cells could maintain PI3K/SGK1 signal transduction.^99^ A similar scenario can be seen in the RAS/RAF/MAPK signaling pathway. Studies have shown that KRAS proto-oncogene (KRAS) mutation, BRAF proto-oncogene (BRAF) V600E mutation, and mitogen-activated protein kinase 1 (MAPK1) gene amplification are involved in EGFR-TKI-resistance.^92,100^ KRAS G12C mutation, KRAS amplification, NRAS proto-oncogene (NRAS) mutations, and MAP2K1 mutations have been detected in lorlatinib-resistant tumors.^56^ KRAS mutations are also associated with GIST resistance to KIT/PDGFR-TKIs.^94^ Furthermore, MEK1 and MEKK1 in-frame deletions have been detected in ROS-TKI-resistant LUAD (lung adenocarcinoma) patients.^101^ A-kinase anchoring protein 9 (AKAP9)-BRAF fusion leads crizotinib resistance.^54^ All of these genes encode key signaling molecules of the RAS/MAPK signaling pathway, and their mutations cause continuous ERK activation and transcription of downstream genes, lead to sustained cell proliferation.

A potential therapeutic approach is to inhibit downstream members of pathway.^102^ For the PI3K/AKT pathway, everolimus, a specific mTOR inhibitor, can block the PI3K/AKT pathway. Several phase II randomized clinical trials have shown that patients with VEGFR-TKI-resistant RCC can take advantage of lenvatinib in combination with everolimus.^103–105^ Everolimus also effectively induces CML cell death in monotherapy or combined with imatinib.^106^ However, one study showed that everolimus has limited efficacy in combination with EGFR-TKIs.^107^ Another clinical study showed no significant clinical benefit of erlotinib in combination with everolimus for neck squamous cell carcinoma (HNSCC).^108^ Consequently, the effectiveness of everolimus in other cancers remains to be explored. The next-generation mTOR inhibitor, Rapalink-1, combined with sunitinib, produced a better therapeutic response in RCC.^109^ PIK3CA inhibitors can theoretically block the PI3K/AKT pathway, but their clinical effectiveness in combination with TKIs was not assessed yet. In addition, direct inhibition of AKTs is also rational. For example, FGFR-TKIs combined with AKT inhibitors can overcome FGFR1 amplification-mediated EGFR-TKI resistance.^110^ AKT inhibitors such as MK2206 and capmatinib can overcome sorafenib resistance in HCC with MET mutations.^111^

For the RAS/MAPK signaling pathway, promising KRAS inhibitors have been introduced in recent years, and some of them, such as sotorasib have shown clinical efficacy.^112^ But the efficacy against TKI resistance is yet undetermined, and the clinical trial of their combination with TKI (NCT04185883) is underway. Fortunately, TKIs combined with RAF inhibitors, MEK inhibitors, or ERK inhibitors have been effective. RAF inhibitors combined with asciminib can treat acute myeloid leukemia (AML) with BCR-ABL-TKI resistance.^113^ The ERK inhibitor, ulixertinib, combined with dasatinib synergistically expedited NB cell death.^114^ Furthermore, the combination of MEK inhibitors and TKIs receives the most attention. MEK inhibitors in combination with TKI is effective in EGFR-TKI-resistant NSCLC cell lines, undifferentiated thyroid cancer (UTC) cell lines, FGFR-TKI-resistant gastric cancer cell lines, NTRK-TKI-resistant multiple tumor cell lines, and EPH receptor A2 (EphA2) TKI-resistant uterine cancer cell lines.^115–119^ However, the efficacy of combination therapy is not supported by all studies.^120^ Clinical trials demonstrated that the MEK inhibitor, trametinib, combined with the multitargeted TKI midostaurin can improve the prognosis of patients with FLT3-TKI-resistant AML.^121^ In addition, P21 (RAC1)-activated kinase 2 (PAK), whose overexpression is related to lenvatinib resistance in some types of TC is also a downstream molecule of RAS. PAK-mediated resistance can be reversed by the combination of PAK inhibitors and lenvatinib.^122^ In vitro studies have shown that dual inhibition of both pathways is an effective approach.^123,124^ The optimal dose is generally determined in phase I clinical trials by balancing efficacy and safety for clinical use.

As mentioned previously, TKIs cannot suppress the sustained activation of pathways due to the downregulation of negative regulators. Mutated in multiple advanced cancers 1 (MMAC1/PTEN) is a typical negative regulator of PI3K/AKT. Its low expression or loss of function is associated with resistance to EGFR-TKIs in LUAD, lapatinib in gastric cancer,^125^ and sunitinib and sorafenib in RCC.^126,127^ Similarly, low expression or loss of dual-specificity phosphatase 6 (DUSP6), a negative regulator of RAS/MAPK, is associated with resistance to FGFR-TKI and EGFR-TKI in lung cancer cell lines.^128,129^ PH domain leucine-rich repeat protein phosphatase (PHLPP), a common negative regulator of PI3K/AKT and MAPK/ERK pathways, is downregulated in EGFR-TKI-resistant NSLCL cell lines.^130^ Incomplete dephosphorylation of signaling molecules due to low expression of negative regulators can lead to TKI resistance. Apart from loss-of-function mutations, epigenetic modifications may be the main reason for the downregulation of negative regulators.

OTU deubiquitinase 1 (OTUD1) is a deubiquitinating enzyme that interacts with PTEN and regulates its stability. Downregulation of OTUD1 can lead to VEGFR-TKI resistance in clear cell renal carcinoma (ccRCC) via the OTUD1-PTEN axis.^131^ Glutaminase 2 (GT2) is also a posttranslational modifying enzyme that induces PTEN degradation, and its high expression is associated with resistance to erlotinib and gefitinib in NSCLC cell lines.^132^ Retinol-binding protein 2 (RBP2) is a histone demethylase that directly downregulates PTEN expression. RBP2 increases CML resistance to TKIs and shifts CML to the acute phase.^133^ In addition, the activated AKT pathway can activate testis-specific Y-encoded-like protein 5 (TSPYL5) and prevent its ubiquitination and subsequent degradation. Upregulation of TSPYL5 inhibits PTEN transcription, leading to drug resistance. Consistently, TSPYL5 inhibition improves drug resistance.^134^ The reasons for the low expression of DUSP1 and PHLPP have not been found, but it was elucidated that high expression levels of miR-452-5p in CRC can negatively regulate DUSP1 and lead to apoptosis resistance, suggesting a role of epigenetics in its regulation.^135^ Thus, low expression of negative regulators can be reversed by epigenetics manipulation.

Additionally, PTPs are physiological antagonists of RTKs, functioning as negative regulators of PTK. Studies have shown that PTPN1 and PTPN2 deficiency in anaplastic large cell lymphoma (ALCL) leads to ALK-TKI resistance in ALCL. PTPN1 is the PTP for ALK and Src homology region 2 containing protein tyrosine phosphatase 2 (SHP2), and oncogenic ALK mutations can inhibit its transcription. Low PTPN1 cannot dephosphorylate SHP2 tyrosine residues, resulting in the overactivation of downstream pathways. Dual blockade of ALK and SHP2 enhances ALK TKI efficacy.^136^ In addition, protein tyrosine phosphatase receptor type O (PTPRO) is a PTP for EGFR and Erb-b2 receptor tyrosine kinase 2 (ERBB2). Low expression of PTPRO enhances EGFR function, leading to lapatinib resistance. The lapatinib sensitivity was restored via upregulation of PTPRO by reducing cellular methylation by 5-azacytidine. It suggests that epigenetics is responsible for PTPRO downregulation.^137^

Interestingly, protein phosphatase 2 (PP2A) is a serine/threonine phosphatase and negatively regulates several signaling pathways. Previously it was shown that the expression levels of cellular inhibitors of PP2A (CIP2A) and novel splice variants of CIP2A are associated with sensitivity to imatinib in myeloid leukemia. Another study reported that decreased CIP2A expression restores the sensitivity of RCC to sunitinib, but the mechanism was unclear and may be related to the overactivation of the control pathway. However, CIP2A levels did not affect the sensitivity to second-generation TKIs such as dasatinib and nilotinib.^138,139^ Therefore, CIP2A levels may contribute to TKI selection.

#### Bypass mechanisms of resistance

As mentioned earlier, several RTKs can activate PI3K/AKT and RAS/MAPK pathways. The efficacy of TKI decreases by simultaneous mutation of several PTKs in tumor cells.^140^ For example, PTKs overactivation mutations other than EGFR mutations such as MET amplification, IGF-1R overexpression, HER2 amplification, AXL overexpression, ROS1 rearrangement, ALK rearrangement, and coiled-coil domain containing 6 (CCDC6)-RET fusion gene exist in EGFR-TKI-resistant cell lines.^92,141–145^ IGF-1R and MET also confer resistance to ALK and VEGFR-targeted therapies.^146–148^ Studying several cancer tissues with ALK-TKI-resistance showed that KIT D820E, MET E1012, EGFR P26_C291del, and MET amplification can lead to crizotinib resistance, and EGFR P753S can lead to alectinib resistance.^149,150^ Moreover, nerve growth factor (NGF)-mediated RAS/MAPK pathway activation can cause resistance to several TKIs in NB.^114^ The upregulation of AXL leads to FLT3-TKI quizartinib resistance in CML and imatinib resistance in GIST.^151,152^ The upregulation of KIT ligand SCF causes ErbB-TKI pyrotinib resistance.^153^ Moreover, EphA2 upregulation induces ccRCC resistance to VEGFR-TKI sunitinib.^154^ Overexpression of EphB family members induces lapatinib resistance in breast cancer cell lines and sorafenib resistance in HCC cell lines.^155,156^ Upregulated receptors or ligands can enhance the signaling, while point mutations and gene rearrangements lead to continuous activation of RTK without ligands, activating the PI3K/AKT and RAS/MAPK pathways and bypassing TKIs. In addition, the crosstalk between different RTKs, such as the crosstalk between MET, EGFR, and HER-3, leads to more complicated abnormal signaling.^157,158^ In addition to co-existing mutation of several PTKs, downstream signaling molecules can conduct compensatory signaling. For instance, upregulation of FOXK2 leads to VEGFA overexpression in UTC. VEGFA binded to VEGFR1 could promote angiogenesis and the transcription of FOXK2, thereby preventing the VEGFR2 inhibition by apatinib.^159^

In addition to RTKs, other receptor signals on the cell membrane or in the cell can activate PI3K/AKT and RAS/MAPK pathways. CD73, a membrane-bound nucleotidase of cancer cells, can activate the AKT pathway and cause lenvatinib resistance in HCC. CD73-related AKT overactivity enhances c-myc function. Subsequently, c-myc promotes SRY-Box transcription factor 9 (SOX9) expression and inhibits glycogen synthase kinase three beta (GSK3β) expression to prevent SOX9 degradation.^160^ The role of SOX9 has not been fully understood. SOX9 overexpression may be associated with stem cell features such as self-renewal and high proliferation capacity. SOX9 overexpression may also induce EMT by activating TGF-β/SMAD pathway.^161^ Moreover, CD73 increases the production of adenosine, an immunosuppressive metabolite that binds to the G protein-coupled adenosine A2a receptor (A2aR) on immune cells, and inhibits the immune response.^162^

Integrins are involved in cellular recognition and adhesion, and link the extracellular stimulus with the intracellular alterations. Integrin-mediated signal transduction has been implicated in many cellular processes. Integrins rely on some NRTKs, such as Src and FAK, to activate the downstream pathways and maintain signal transduction. Osteopontin (OPN) promotes tumor cell proliferation by binding integrin avβ3 and activating downstream FAK/AKT and ERK signaling pathways. Overexpression of OPN in NSCLC treated with EGFR-TKI compensated for the blockade of proliferation signals, causing EGFR-TKI-resistance.^163^ In the Ph+ CML cell lines, integrin β3 activated integrin-linked kinase (ILK) in response to extracellular signals, leading to imatinib resistance.^164^

Numerous cytokines and chemokines in TME can bind to their receptors on the cancer cell membrane and activate their downstream pathways. It was revealed that interleukin 6 (IL-6) leads to osimertinib resistance by binding to its receptors and activating LAMA5/FAK signaling.^165^ Tumor necrosis factor (TNF) is a mediator of intrinsic resistance to EGFR-TKIs. TNF binds to TNFR and activates the c-Jun N-terminal kinase (JNK) to increase growth arrest-specific 6 (Gas6) expression, which binds to AXL, activates ERK signaling, and resists EGFR inhibition. Disruption of the TNF-JNK-Axl-ERK axis at any level increases the sensitivity of GB to EGFR-TKIs.^166^ In CRC, CCR2 activates PI3K/AKT/GSK3β signaling and maintains β-linked protein stability, leading to regorafenib resistance.^167^ Ribonucleotide reductase subunit M2 (RRM2) competes with ubiquitin-protein ligase E3A (UBE3A) in RCC to bind to ANXA1, prevent ANXA1 degradation, and activate the AKT pathway, resulting in sunitinib resistance.^168^ In addition, Src, as an NRTK, also activates the PI3K/AKT pathway, leading to ALK-TKIs resistance in NSLCL.^169^

The JAK/STAT pathway is also a downstream of PTKs. It was shown that NGF/ tropomyosin receptor kinase A (TrkA) can alternatively activate the JAK/STAT3 pathway to induce EMT and erlotinib resistance in HNSCC.^170^ The Pim-1 proto-oncogene (PIM) is a downstream of JAK/STAT pathway. High PIM expression is associated with ALK-TKIs resistance in NB.^171^ Simultaneous inhibition of several signaling molecules in the JAK/STAT pathway could effectively overcome drug resistance.^171–173^

The combination of TKIs and inhibitors of downstream molecules or chemotherapeutic agents can effectively mitigate drug resistance.^174^ Administration of multitargeted TKIs and combinations of different TKIs are more effective. For instance, cabozantinib is an oral multikinase inhibitor targeting VEGFR1/2/3, MET, and AXL and has an acceptable efficacy in patients with advanced HCC and GIST.^175,176^ Repotrectinib is a next-generation ROS1/TRK/ALK multitarget inhibitor that effectively treats crizotinib-resistant lung cancer with ROS1 rearrangements. It also effectively treats brain metastases of lung cancer, thanks to its ability to cross the blood-brain barrier.^177^ Lenvatinib contributes to overcoming sorafenib resistance in HCC with FGFR4 expression.^178^ Gilteritinib is an FLT3/AXL-TKI that can treat ALK-TKI-resistant NSCLC and AML with FLT internal tandem duplication (ITD).^179,180^ Entrectinib is a multitargeted TKI that has been recently approved for multiple solid tumors with ROS fusion genes and NTRK1/2/3 fusion genes.^181^

The combination of TKIs has also been promising. Herein, the combination of gefitinib and apatinib has been more effective in NSCLC cell lines compared with each separately.^182^ Gefitinib combined with IGF1R-TKI may be effective against GBM with IGFR activation.^183^ Several MET-TKI and EGFR-TKI combinations have been effective in NSCLC patients with MET amplification and drug-resistant mutations.^184,185^ Lenvatinib in combination with gefitinib improves lenvatinib resistance in patients with HCC.^186^ FAK-TKI defactinib (VS-6063) could restore gefitinib sensitivity in gefitinib-resistant cell lines by blocking the downstream pathway.^187^ The triple combination of repotrectinib, EGFR-TKI, and MEK inhibitor was effective in cancer cells with NTRK1-G595R resistance, aberrant EGFR activation, and ERK reactivation.^188^ Some drugs other than TKIs can inhibit PTK. For instance, aspirin inhibits IGF-1R. Regorafenib, in combination with aspirin, had similar efficacy. As an anti-inflammatory agent, aspirin also alleviated inflammation.^189^ Berberine is a natural MET inhibitor with similar efficacy to MET-TKIs, which can be used in combination with TKIs.^190^

Interestingly, PTK can be indirectly targeted. Heat shock protein 90 (HSP90) acts as a molecular chaperone, maintaining the stability of RTKs (HER2, KIT, MET, etc.), AKT, ERK, and other signaling proteins. Therefore, HSP90 may indirectly inhibit PTK and other signaling molecules. In GIST cell lines with aberrant KIT activation and imatinib resistance, HSP90 is necessary for proper KIT folding, and HSP90 inhibitor TAS-116 reduces autophosphorylation-activated KIT and inhibits tumor growth.^191^ HSP90 inhibition combined with lapatinib has also been effective in breast cancer.^192^

HGF binds to MET after MET has been integrated with GRB2-associated binding protein 1 (Gab1). Metformin prevents MET from integrating with Gab. Studies have shown that metformin combined with alectinib may improve HGF/MET-induced resistance to alectinib in NSCLC cell lines.^193^ TKI, in combination with chemotherapy, is also effective. Crizotinib, combined with CHOP chemotherapy regimens, had a strong synergistic effect on ALCL in animal models and prevented drug resistance.^194^

#### Nuclear translocation

Nuclear translocation is a subcellular process and a part of the signal transduction pathway. Activated cytoplasmic proteins are transported to the nucleus to alter gene expression in response to external stimuli.^195^ Membrane EGFR translocation to the cytoplasm/nucleus is involved in resistance to gefitinib and osimertinib. EGFR translocation is associated with inhibition of the Hippo pathway, which may be linked to TKI resistance.^196^ Hippo signaling pathway prevents cell growth by sensing upstream stimuli and inhibiting the transcriptional function of YAP.^197^ Mechanistically, TKI can stimulate membrane EGFR (mEGFR) to translocate into the cytoplasm. By altering the molecular interaction of the central kinase complex of the hippo pathway, cytoplasmic EGFR (cEGFR) liberates YAP from cytoplasmic sequestration and increases its nuclear translocation. cEGFR can also accompany YAP into the nucleus. Nuclear EGFR (nEGFR) and YAP increase the transcription of downstream target genes, leading to cell proliferation and drug resistance.^196^

TIP30 is a tumor suppressor that negatively regulates both PI3K/AKT and RAS/MAPK pathways in NSCLC and prevents EGFR nuclear translocation from inhibiting nEGFR-mediated c-myc transcription. Low expression of TIP30 is associated with gefitinib resistance in NSCLC cell lines, while upregulation of TIP30 improves drug resistance by attenuating EGFR signaling pathway.^198^ In NSCLC cell lines, TKI-bounded EGFR can dimerize with other RTKs like HER and AXL to promote nuclear translocation of protein kinase C δ (PKCδ), a member of the PKC family.^199^ Nuclear PKCδ phosphorylates key nuclear proteins to regulate apoptosis. The intranuclear enrichment of PKCδ in gefitinib-resistant NSCLC promotes cell survival due to the transcription of anti-apoptotic molecules.^200^ The combination of sotrastaurin, a PKC inhibitor, and gefitinib significantly restored tumor sensitivity to gefitinib. However, the exact mechanism needs to be further investigated.

Although most of the previous studies have focused on typical RTK signaling pathways neighboring the cell membrane, recent findings suggest that at least 12 families of RTKs can be transferred to the nucleus, referred to as “membrane receptors in the nucleus (MRINs)”. High nuclear levels of MRINs are associated with poor prognosis and possess novel noncanonical functions in transcriptional regulation, cell proliferation, and malignant transformation.^201^ The nuclear translocation of EGFR family members contributes to drug resistance. Therefore, it is crucial to determine the mechanisms of nuclear translocation to overcome drug resistance.

In general, TKI resistance due to abnormal signaling pathways is not just attributed to abnormal membrane receptors. Abnormal activity and localization of signaling molecules and their regulatory molecules can also lead to sustained activation of the pathway. In addition, epigenetic modifications can affect the expression of signaling molecules, and recognition of these epigenetic alterations contributes to developing new drugs (Table 2).

**Table 2:** Clinical studies of TKI in combination with other drugs to reverse drug resistance

Brief information of RCT				Clinical Validity	Grade 3/4 treatment-related adverse events	
TKI	Co-drugs	NCT number (phase)	Experimental VS Controlling	Main indicators(month)	AE incidence	Major AE
**Non-small-cell lung carcinoma**						
Erlotinib	Ramucirumab (anti-VEGFR)	NCT02411448 (III)	Erlotinib + Ramucirumab (224) VS Erlotinib + Placebo (225)	PFS: 19.4 VS 12.4	72% VS 54%	Hypertension (24%) Dermatitis acneiform (15%)
Erlotinib	Bevacizumab (anti-VEGFR)	UMIN000017069 (III)	Erlotinib + Bevacizumab (114) VS Erlotinib alone (114)	PFS: 16.9 VS 13.3 OOR: 72% VS 66%	88% VS 46%	Rash (21%)
Gefitinib	Capmatinib (MET-TKI)	NCT01610336 (Ib/II)	Capmatinib + Gefitinib	ORR: Cross phase:27% In MET copy > 6: 47%	–	Increased amylase and lipase levels (6%)
Osimertinib	Savolitinib (MET-TKI)	NCT02143466 (Ib)	B1: Previous 3^rd^ EGFR TKI (69)	PFS: 5.4; OOR: 30% DOR: 7.9	57%	
			B2: No previous 3^rd^ EGFR TKI, T790M-(51)	PFS: 9; OOR: 65% DOR: 9.0		
			B3: No previous 3^rd^ EGFR TKI, T790M^+^ (18)	PFS: 11; OOR: 67% DOR: 12.4	–	–
			D: No previous 3^rd^ EGFR TKI, T790M^+^ (42). Half dosage of Savolitinib in B.	PFS:9.1; OOR: 64% DOR: 8.0	38%	–
Afatinib	Cetuximab (anti-EGFR)	NCT02438722 (II)#	Afatinib + Cetuximab (83) VS Afatinib alone (85)	PFS*: 11.9 VS 13.4	72% VS 40%	Acneiform rash (27%) Maculopapular rash (13%) Diarrhea (15%)
Osimertinib	Selumetinib (MEK1/2i)	NCT02143466 (Ib)	Osimertinib + Selumetinib (36)	OOR: 42%	58%	
	Savolitinib (MET-TKI)		Osimertinib + Savolitinib (18)	OOR:44%	600 mg 75% 800 mg 83.3%	–
	Durvalumab (anti-PD-1)		Osimertinib + Durvalumab (23) #	OOR: 43%	3 mg/kg 60% 10 mg/kg 38.5%	–
Osimertinib	Bevacizumab (anti-VEGFR)	NCT03133546# (II)	Osimertinib + Bevacizumab VS Osimertinib alone	PFS*: 15.4 VS 12.3 OS:24 VS 24.3 OOR:55%VS 55% TTF: 8.2VS10.8	47% VS 18%	Hypertension (24%) Lipase increased (7%)
Gefitinib	Fulvestrant (antiestrogen)	NCT01556191# (II)	Gefitinib + Fulvestrant (100)VS Gefitinib alone (104)	PFS*: 9.9 VS 9.4 OS*: 22.1 VS 28.6	21.4%VS 24.2%	Investigations (8.7%) Skin and subcutaneous tissue disorders (6.1%)
Erlotinib			Erlotinib+ Fulvestrant (88) VS Erlotinib alone (87)	PFS*: 1.8 VS 2.0 OS*: 10.3 VS 7.3	13.8% VS 15.9%	Gastrointestinal disorders (3.4%)
Osimertinib	Ramucirumab (anti-VEGFR)	NCT02789345 (I)	Osimertinib + Ramucirumab	PFS: 11 OOR: 76% DOR: 13.4	28%	Hypertension (8%) Platelet count decreased (16%)
Gefitinib	Pemetrexed (chemo)	- (II)	Gefitinib + Pemetrexed (126) VS Gefitinib alone (65)	PFS:16.2 VS 11.1 PFS in different TS level: High TS (12.6 VS 9.9) Low TS (22.6 VS 11.0) OS: 43.3 VS 36.8	25.4% VS 9.2%	Fatigue (5.6%) Anorexia (4.0%) Mucositis oral (4.0%)
Gefitinib	Carboplatin plus pemetrexed (chemo)	UMIN000006340 (III)	Gefitinib + chemotherapy (170) VS Gefitinib alone (172)	PFS:20.9 VS 11.2 OS: 50.9 VS 38.8 ORR: 84% VS 67%	65.3% VS 31%	Neutropenia (31.2%) Leukopenia (21.2%) Anemia (21.2%) Thrombocytopenia (17.1%)
Osimertinib	Durvalumab (anti-PD-1)	NCT02143466 (Ib)	A (previous TKI treated): Osimertinib + Durvalumab (23)	OOR: 43% DOR:20.4 m	Durvalumab 3 mg/kg:60% 10 mg/kg:38%	Both Interstitial lung disease
			B: Osimertinib + Durvalumab (11)	PFS:6 OOR: 82% DOR:7.1 m	82%	Interstitial lung disease (27%)
Gefitinib	S49076 (MET/AXL TKI)	- (I)#	Gefitinib+S49076(14)	limited anti-tumor activity		
Osimertinib	Carboplatin- pemetrexed (Chemo)	jRCTs071180062 (II)#	Osimertinib + carboplatin-pemetrexed VS Single osimertinib	PFS*: 14.6 VS 15.8 OS: NR OOR*: 53.6% VS 71.4%	83.8%VS 45.2%*	Neutropenia (51.6%) Leukopenia (38.7%
Gefitinib	Vorinostat (HDACi)	NCT02151721 (I)	Gefitinib + vorinostat (12)	PFS: 5.2 OS: 22.4 DOR:83.3%	50%	Hypokalemia (16%)
Apatinib	Pemetrexed –platinum (Chemo)	ChiCTR1800015920 (II)	Apatinib + Pemetrexed platinum (20)	PFS: 7.7 OS: 20.1 OOR: TKI-pretreated:90% TKI naive:70%	35%	Neutropenia (25%)
Afatinib	Anti-VEGF vaccination (anti-EGFR)	NCT03623750 (Ib)	Apatinib + anti-EGF vaccination (23)	PFS: 14.8 OS: 26.9 OOR: 78.30% DOR:13.7	30%	Diarrhea (26%)
Alectinib	Bevacizumab (anti-VEGFR)	NCT04181060 (I/II)	Initial treatment: Alectinib + Bevacizumab (6)	PFS: NR OS: 9 OOR: 100% DCR:100%	–	–
			Prior ALK TKI treatment: alectinib +bevacizumab (5)	PFS: 9.5 1 year OS rate: 63.6% OOR: 60% DCR:100%	27%	Hypertension (9%) Proteinuria (9%)
Erlotinib	Nivolumab (anti-PD-1)	NCT01454102 (I)	Erlotinib + nivolumab (21): TKI-treated (20) TKI-naive (1)	PFS: 5.1 OS:18.7 1 year OS rate:70% OOR: 15%	52.4%	Diarrhea (10%) AST increased (10%)
Tepotinib	Gefitinib (TKI)	NCT01982955 (Ib/II)	Tepotinib + Gefitinib (31)	PFS:17.3 OS: 17.3 OOR: 45%	62%	Amylase concentration increased (16%) Lipase concentration increased (13%)
			Chemo (24)	PFS:18.7 OS: 18.7 OOR: 33%	52%	Anemia (30%) Neutrophil count decreased (13%)
Renal Cell Carcinoma						
Axitinib/Sunitinib	Pembrolizumab (anti-PD-1)	NCT02853331 (III)	Pembrolizumab + Axitinib (432) VS Sunitinib (429)	PFS: 15.1 VS 11.1 OS: 89.9% VS 78.3% OOR: 59.3% VS 35.7%	75.8% VS 70.6%	Hypertension (22.1%) ALT decreased (13.3%)
Lenvatinib/ sunitinib	Pembrolizumab (anti-PD-1) Everolimus (mTORi)	NCT02811861 (III)	Lenvatinib +Pembrolizumab (355)	PFS: 23.9 1 year OS rate: 79.2% OOR: 71.00% DOR:25.8 m	82.40%	Hypertension (27.6%) Diarrhea (9,7%)
			Lenvatinib + Everolimus (357)	PFS: 14.7 1 year OS rate: 66.1% OOR: 53.50% DOR:16.6 m	83.10%	Hypertension (22.5%) Diarrhea (11.5%)
			Sunitinib (356)	PFS: 9.2 1 year OS rate: 70.4% OOR: 36.10% DOR: DOR:14.6%	71.80%	Hypertension (18.8%)
Lenvatinib	Everolimus (mTORi)	NCT01136733 (II)	Lenvatinib + Everolimus (51)	PFS: 14.6 OS: 24 OOR: 43%	71%	Constipation (37%) Diarrhea (20%)
			Single lenvatinib (52)	PFS: 7.4 OS: 19.1 OOR: 27%	79%	Hypertension (17%) Diarrhea (12%)
			Single everolimus (50)	PFS: 5.5 OS: 15.4 OOR: 6%	50%	Anemia (12%) Hypertriglyceridaemia (8%)
Lenvatinib	Everolimus (mTORi)	NCT02915783 (II)	Lenvatinib +everolimus (31)	PFS: 9.2 OS: 15.6 OOR: 26%	68%	Hypertension (16%) Malignant progression (13%)
VEGFR-TKI	Everolimus (mTORi)	NCT01266837 (IV)	Everolimus (63)	PFS: 3.8 OS: 16.8 OOR: 7.90% DOR: 60.3%	57.10%	Anemia (17.5%) hyperglycaemia(7.9%) fatigue(4.8%)
Hepatocellular Carcinoma						
Lenvatinib Hcc	Pembrolizumab (anti-PD-1)	NCT03006926 (Ib)	Lenvatinib + Pembrolizumab (104)	PFS: 9.3 OS: 22 OOR: 46% DOR:8.6	67%	Hypertension (17%) AST increased (11%)
Sorafenib	Trametinib (MEK1/2i)	NCT02292173 (I) #	Sorafenib + Trametinib (17)	PFS: 3.7 OS: 7.8	–	Elevated AST (37%) hypertension (24%)
Cabozantinib (multi-target TKI)		NCT01908426 (III)	Cabozantinib (407) VS placebo (237)	PFS: 5.2 VS 1.9 OS:10.8 VS 8 OOR: 4%	68% VS 36%	Palmar-plantar erythrodysesthesia (17%) hypertension (16%) increased ALT (12%)
Endometrial Carcinoma						
Lenvatinib	Pembrolizumab (anti-PD-1)	NCT02501096 (II)	Lenvatinib +Pembrolizumab (108)	PFS: 7.4 OS: 16.7 OOR: 63.6 DOR 21.2	66.90%	Hypertension (31.5%) fatigue (7.3%) diarrhea (6.5%)
Lenvatinib	Pembrolizumab (anti-PD-1)	NCT03517449 (III)	Lenvatinib + Pembrolizumab (411) VS Chemo (416)	PFS: 7.2 VS 3.8 OS: 18.3 VS 11.4	88.9% VS 72.7%	Hypertension (37.9%) weight decrease (10.3%)
Breast Cancer						
Tucatinib	Ado-Trastuzumab Emtansine (TDM1) (anti-HER2)	NCT01983501 (Ib)	Tucatinib +T-DM1(57)	PFS: 8.2 OOR: 47% DOR:6.9	52%	ALT increased (12%) AST increased (12%)
Cervical Cancer						
Apatinib	Camrelizumab (anti-PD-1)	NCT03816553 (II)	Apatinib + Camrelizumab (45)	PFS: 8.8 OS:NR OOR: 55.60% DMR:NR	71.10%	Hypertension (24.4%) anemia (20%)
Head And Neck Squamous Cell Carcinomas						
Erlotinib	Everolimus (mTORi)	NCT00942734 (II)	Erlotinib + Everolimus (35)	PFS: 2.9 OS: 10.3 OOR: 2.80%	–	–
Multiple Solid Tumors						
Afatinib	Sotorasib (RAFi)	NCT04185883 (I)	Recruiting			
Lung Adenocarcinoma						
Osimertinib	Bevacizumab (anti-VEGFR)	UMIN000023761 (II)	Osimertinib + Bevacizumab VS Single osimertinib	PFS*: 9.4 VS 13.5 OS*: NR VS 22.1 OOR: 68% VS 53%	80%	Proteinuria (23%) hypertension (20%)
Chronic Myeloid Leukemia						
imatinib CML	HCQ (Autophagy inhibitor)	NCT01227135 (II)	Imatinib + HCQ (30) VS Imatinib (32)	Success Rate (BCR-ABL1 qPCR level reduction≥0.5 log after 12 m): 12 m: no difference; 24 m: 20.8% higher than single imatinib MMR: 12 m (66.7% VS 71.9%); 24 m (63.3% VS 68.8%). CMR: 12 m (0% VS 0%); 24 m (3.3% VS 6.3%).		
Gastrointestinal Stromal Tumor						
Cabozantinib (multi-target TKI) GIST		NCT02216578 (II)	Cabozantinib (50)	PFS: 5.5 OS: 18.2 OOR: 60% DOR: 82%	68%	Hypertension (36%) diarrhea (26%)

*No statistical difference. #: The trial shows limited clinical benefit or no benefit from combination therapy.

chemo: chemotherapy; MEK1/2: mek1/2 inhibitor; mTORi: mTOR inhibitor; HCQ: hydroxychloroquine; HDACi: histone deacetylases inhibitors; TDM1: trastuzumab emtansine; RAFi: RAF inhibitor; PFS: progression-free survival; OS: overall survival; ORR: objective response rate; DOR: duration of response; DMR: deep molecular response; TTF: time to treatment failure; AE: adverse event; NR: not reached; MMR: major molecular response; CMR: complete molecular response; TS: thymidylate synthase.

### Tumor microenvironment

**Fig. 3:**
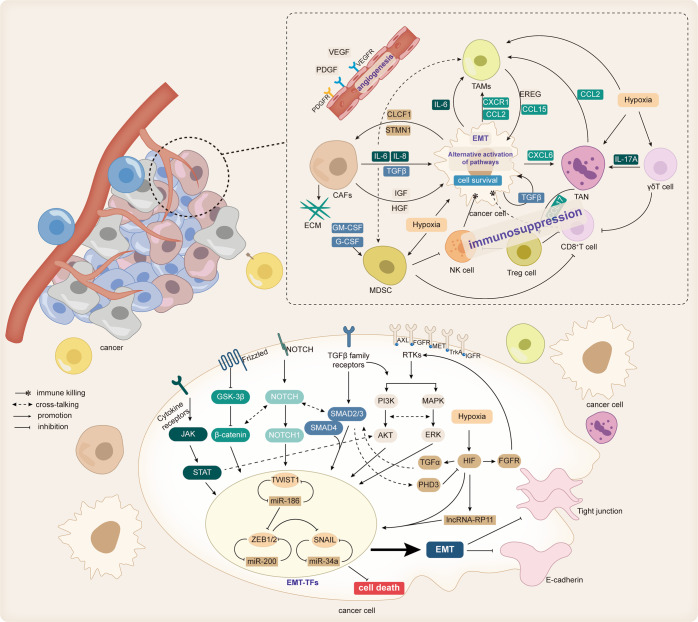
Cellular composition within the TME and the effect of EMT on TKI resistance. Cells within the TME include tumor cells, cancer-associated stromal cells, and immune cells. These cells crosstalk with other cells through cytokines and chemokines, promoting tumor progression. Some cell-secreted ligands, such as TGF-β, IL-6, HGF, IGF, etc., can bind to the corresponding receptors on the surface of tumor cells and affect the biological behavior of tumor cells, one of which is EMT. EMT can be mediated by the TGFβ/Smad classical pathway. Some cytokine receptors, such as IL-6 and some RTKs (AXL, FGFR, MET, IGFR, TrkA, etc.) can also activate the PI3K/AKT, RAS/MAPK, and JAK/STAT pathways in combination with the corresponding ligands to induce EMT. The Notch signaling pathway and wnt/β-catenin pathway are also involved in EMT, and there is crosstalk between these pathways. Tumor cells that develop EMT have a more aggressive phenotype and metastatic ability and are resistant to cell death and TKI resistance. In addition, the immunosuppressive state within the TME impairs the killing of tumor cells by immune cells and promotes tumor survival

TME is enormously complex and includes various cell types, signaling molecules, cytokines, extracellular matrix (ECM), and blood vessels. TME is characterized by hypoxia, immunosuppression, and chronic inflammatory response. TME interacts with tumor cells, supports tumor cell proliferation and survival, and influences therapeutic response by modulating biological behaviors, such as tumor cell growth and proliferation. TKI therapy may induce TME remodeling and improve therapeutic response, but TME alterations can also induce drug resistance and maintain tumor progression. Uncontrolled expression of bioactive substances by cellular components of TME can compensate for suppressed signal transduction in tumor cells, induce immunosuppression, and promote epithelial-mesenchymal transition (EMT) and stemness of tumor cells, all of which are closely related to TKI resistance. In addition, the dysregulated metabolism of cellular components of TME can also affect cancer cells and promote TKI resistance (Fig. 3).

#### Cellular component

Cellular components of TME include tumor cells and resident components, and recruited host cells known as cancer-associated stromal cells and immune cells. These cells promote tumor progression and induce drug resistance by releasing numerous cytokines and chemokines. Cancer-associated fibroblasts (CAFs), tumor-associated macrophages (TAMs), tumor-associated neutrophils (TANs), regulatory T cells (Tregs), and myeloid-derived suppressor cells (MDSCs) are among these cells, and play important roles in the development of TKI resistance.

CAFs can release various cytokines, suppress immune function, shape a drug-resistant microenvironment, and interact with tumor cells. CAFs are of great importance for developing drug resistance.^202^ CAFs-derived HGF and IGF-1 conduct primary resistance to TKIs targeting different oncogenes.^203^ CAFs in osimertinib-resistant NSCLC cell lines release higher IL-6, IL-8, and HGF and express more CAF markers like α-smooth muscle agonists-α (α-SMA), fibroblast activation protein (FAP), and PDGFR.^204^ The same scenario can be seen in patients with metastatic RCC.^205^ HGF can bind to MET on cancer cells to activate MEK/ERK signaling pathway. IL-6 interacts with transforming growth factor-beta (TGF-β) to induce EMT.^206,207^ Through the PI3K/Akt/hypoxia-inducible factor 1 subunit alpha (HIF-1α) signaling pathway, IL-8 upregulates forkhead box C1 (FoxC1) expression to promote the expression of C-X-C motif chemokine receptor 1 (CXCR-1) and C-C motif chemokine ligand 2 (CCL2) to induce TAM infiltration and immunosuppression. In HCC, hepatic stellate cell (HSC)-derived CAFs release more HGF, which activates MET pathway and increases STMN1 expression in HCC cells. STMN1 promotes PDGF-BB expression, inducing HSC to acquire CAF characteristics and further elevate HGF level.^208^ On the contrary, HCC cells release B-cell activating factor (BAFF) to bind BAFF receptors on CAFs, activate the NF-κB pathway, and increase the expression of IL-6 and IL-8, causing sorafenib resistance.^209^ Apart from HGF, CAFs can also secrete IGF-1, which binds to IGF-1R on cancer cells, thereby promoting Annexin A2 (ANXA2) expression.^210^ The exact mechanism underlying ANXA2 effects was not uncovered. The current evidence suggests that ANXA2 plays a role in EMT and ECM degradation.^211^

CAFs-derived cardiotrophin-like cytokine factor 1 (CLCF1) stimulates HCC cancer cells to release TGF-β and C-X-C motif chemokine ligand 6 (CXCL6). TGF-β induces the stem cell characteristics, and CXCL6 induces N2-like polarization of TANs, forming a TME proper for cell stemness and immunosuppression. Meanwhile, CXCL6 induces more CLCF1 secretion, forming a positive feedback loop.^212^ Moreover, CAFs in luminal-like HER2^+^ breast cancer cell lines can release NRG1β, which binds to HER3 and stabilizes the HER2-HER3 dimer to maintain everlasting activation of downstream pathways, resulting in lapatinib resistance.^213^ In refractory tumors, CAFs promote tumor angiogenesis and progression despite administrating VEGFR TKIs,^214^ suggesting the existence of VEGFR-independent PDGFR-related angiogenesis pathways. Moreover, pericytes may contribute to this observation by increasing pericyte coverage and pericyte-derived VEGF.^215^

TAMs are abundant in the TME and are divided into two subgroups, M1 and M2. Tumor cells can promote TAM M2 polarization during treatment, which provokes drug resistance through multiple mechanisms. By secreting VEGF and IL-6, HNSCC cancer cells recruit macrophages and promote the M2 polarization. M2-polarized TAMs can secret CCL15 to bind C-C motif chemokine receptor 1 (CCR1) on tumor cells and activate the NF-κB pathway, thereby inducing gefitinib resistance.^216^ Metformin, an inhibitor of CCL15 expression, maintains gefitinib sensitivity. Moreover, high TAM-derived epiregulin (EREG) induces the formation of EGFR/ERBB2 heterodimers on cancer cells, induces AKT phosphorylation, and attenuates TKI-induced apoptosis, thereby reducing erlotinib therapeutic response in NSCLC patients.^217^ However, overexpression or knockdown of cancer cell-derived EREG does not affect the TKI sensitivity, indicating the importance of compensatory signaling pathways in TKI resistance. Upregulation of TAM-derived EREG may attribute to the activation of the JNK pathway by IL-1β in the TME.^218^ In erlotinib-resistant HNSCC, EREG/EGFR was found to upregulate the c-myc expression to stimulate cell proliferation.^219^ Furthermore, M2 TAMs can release HGF to bind to MET receptors in HCC tumor cells and induce sorafenib resistance. It also helps recruit more TAMs from the circulation to TME, deteriorating the situation.^220^

Similar to TAMs, TANs can also be polarized in response to TME signaling, and N2 TANs contribute to tumorigenesis. In an animal study, a high dose of VEGF-TKI induced γδ T cells to secrete IL-17A. IL-17A contributed to the N2 polarization of TANs and depletion of CD8^+^ T cells, forming an immunosuppressive microenvironment, resulting in VEGFR-TKI resistance.^221^ Similarly, sorafenib treatment for HCC was accompanied by a progressive increase in TAN infiltration, and depletion of TAN reversed sorafenib resistance. Mechanistically, sorafenib-related antiangiogenic therapy-induced hypoxia-activated HIF-1α/NF-κB signaling increases chemokine CXCL5 secretion in HCC cells, thereby recruiting TAN and inhibiting apoptosis. TANs further provoked TAM and Treg cell infiltration by releasing CCL2 and CCL17. These alterations weakened immune response but stimulated angiogenesis, making tumor cells insensitive to VEGFR-TKIs.^222^

Treg cells, which express CD25 and forkhead box P3 (FOXP3), induce immunosuppression, antigen tolerance, and immune evasion. In HCC, CCL22, which can induce CCR4^+^ Treg cells migration into the TME, was upregulated under sorafenib administration. Its upregulation was mediated by TNFα-related receptor-interacting serine/threonine-protein kinase 1 (RIPK1)/NF-κB pathway,^223^ and TGF-β can also upregulate CCL22 through TGF-β-miR-34a-CCL22 axis.^224^ Upregulating T-cell factor 1 (TCF1), programmed cell death protein 1 (PD-1), and cytotoxic T-lymphocyte-associated protein 4 (CTLA-4) and releasing immunosuppressive cytokines like IL-10, Treg cells convey sorafenib resistance through an immunosuppressive landscape. Under such conditions, CCL22 or CCR4 blockade reduces the Treg population and improves sorafenib resistance.^225^ CCR4 blockade may be more effective because CCL17 also activates CCR4.^223,224^

MDSCs proliferation can be induced by cytokines and chemokines, and activated MDSCs enhance angiogenesis and suppress T-cell function by secreting various cytokines, playing a pro-tumorigenic and immunosuppressive role.^226^ In RCC, sunitinib can strongly inhibit MDSCs in the peripheral blood and spleen, but MDSCs in TME are highly resistant to sunitinib.^227^ Mechanistically, despite the partial inhibition of MDSCs’ proliferation ability through sunitinib-mediated STAT3 inhibition, MDSCs can maintain their proliferation in the presence of proliferative stimuli such as IL-6, granulocyte-macrophage colony-stimulating factor (GM-CSF), and granulocyte colony-stimulating factor (G-CSF),^228^ among which disordered GM-CSF can bypass the sunitinib-mediated STAT3 inhibition through STAT5 activation, providing alternative survival signals for MDSCs, exacerbating the immunosuppression, and increasing VEGF expression, leading to VEGFR-TKIs resistance. A novel role of MDSCs was found in EGFR-mutated LUAD. A subset of MDSCs-CD14^+^S100A9^+^ monocytic MDSCs differentiated to TAM. S100A9^+^ MDSCs-derived TAMs, which express S100A9^+^ and M2 marker CD206, attenuate the cytotoxic effect of EGFR-TKIs.^229^ Novel RELB alternative NF-κB pathway activation may be the reason, whose function in cell cycle arrest and death inhibition was shown by previous studies.^230,231^ In HCC, MDSCs-derived IL-6 were found to induce HCC cells to secret FGF-1. FGF-1 can activate CAFs and induce sorafenib resistance,^232^ suggesting the crosstalk between cellular components. These findings show the interaction in different cell types.

Cellular components of TME can aggravate immunosuppression, necessitating combination therapy with TKI and immune checkpoint inhibitors (ICIs). Theoretically, ICIs alleviate the immunosuppressive microenvironment and promote antitumor immune responses. TKIs can also activate immune cells, and there is no significant overlap in the toxicity of TKIs and ICIs.^233^ A phase III clinical trial showed that axitinib combined with pembrolizumab significantly prolonged the survival of RCC patients, compared with sunitinib alone.^234^ Clinical trials of lenvatinib combined with pembrolizumab for endometrial cancer, HCC, and RCC have also shown encouraging safety and efficacy.^235–239^ Lapatinib combined with camrelizumab, erlotinib combined with nivolumab, and osimertinib combined with bevacizumab have been promising in NSCLC.^240–242^ But nivolumab combined with EGFR-TKIs had a higher incidence of interstitial pneumonia although effectively treated NSCLC.^243^ However, despite improving immune function, lenvatinib combined with anti-PD-1 did not lead to durable tumor regression in TC,^244^ and these findings need to be validated with further studies.

To sum up, extensive interactions between various cellular components of TME contribute to tumorigenesis and immunosuppression. TKIs, especially anti-angiogenesis-related TKI (VEGFR-TKI, PDGFR-TKI, etc.), profoundly modulate TME to undermine tumor cell viability, but TME remodeling sometimes promotes drug resistance. The immunosuppressive TME markedly promotes drug resistance and tumor progression. Removing an immunosuppressive TME may attenuate cellular component-mediated TKI resistance.

#### Histological phenotypic transformation

EMT is a reversible process of the trans-differentiation of polarized epithelial cells to mesenchymal cells mediated by EMT transcription factors (EMT-TFs), such as snail family transcriptional repressor (SNAIL), zinc finger e-box binding homeobox (ZEB), and twist family BHLH transcription factor (TWIST). The process is associated with the removal of epithelial cell markers like E-cadherin and upregulation of mesenchymal cell markers such as N-cadherin and vimentin. In this process, tumor cells gradually lose their epithelial cell phenotype and intercellular adhesion junctions. Then, they obtain stem cell-like characteristics and possess greater mobility and migration ability.^245,246^ TKI-induced TME remodeling can induce abnormal activation of intracellular EMT-TFs,^247^ leading to the development of EMT, and promoting tumor progression, metastasis, and resistance to TKIs.

The mechanism of TKI resistance mediated by EMT is complex. Recent findings suggested that EMT may be an independent resistance mechanism. High ZEB1 expression without resistance mutations has been reported in erlotinib-resistant NSCLC cells, and knockdown of ZEB1 may restore the erlotinib sensitivity to the same level as the parental cells.^248^ A similar scenario has been observed in NSCLC cell lines resistant to gefitinib or osimertinib.^249^ Furthermore, EMT-related molecules such as SNAIL 1/2, ZEB 1/2, Claudin 1 (CLDN1), and CD44 were remarkably upregulated in tumor biopsies from sunitinib-resistant ccRCC patients.^250^ Moreover, dovitinib-treated surviving cell count in bladder cancer cell lines positively correlated with EMT level (p = 0.0483).^244,251^ These findings suggest that EMT can induce tumor cell resistance to TKI-induced apoptosis. Highly expressed EMT-TFs such as ZEB1 and TWIST1 can directly bind to the intron region and promoter of BIM, in mesenchymal NSCLC cancer cells and repress its transcription.^248,252^ In addition, EMT-TFs can also activate the ATR serine/threonine kinase (ATR)- checkpoint kinase 1 (CHK1)-AuroraB signaling cascade, and Aurora B can phosphorylate and degrade BIM.^253^ In the absence of BIM, TKIs cannot induce apoptosis despite the effective inhibition of oncogenic pathways.

The precipitating factor of EMT may be also associated with TKI resistance. TGF-β is a typical inducer of EMT, and its level increases with increasing gefitinib concentrations in NSCLC,^254^ a feedback regulation that promotes EMT and EGDR-TKI resistance established. Mechanistically, TGF-β binds to TβR (TGFR) to induce EMT-TFs expression to downregulate epithelial-related proteins and initiate EMT via the SMAD pathway or non-SMAD pathways such as PI3K/AKT and RAS/MAPK pathway.^246^ In addition, TGF-β can also induce EMT by inducing several EMT-triggering signaling pathways, such as the Notch, Wnt, and integral protein signaling pathways. Moreover, TGFβR/SMAD pathway downregulates phosphohydroxylase 3 (PHD3). PHD3 can be also downregulated by promoter methylation. PHD3 is a negative regulator of EMT, whose depletion stabilizes HIF-1/2 to upregulate the EGFR ligand TGFα. PHD3 downregulation also enhances EMT and spontaneous metastasis. In return, TGFα stimulates EGFR, thereby potentiating SMAD signaling, and reinforcing EMT and metastasis.^255^ Besides, studies in GB have shown that TGF-β also promotes YAP nuclear translocation and upregulates SNAIL2.^256^

Hypoxia is also a key trigger of EMT, which relies on HIF to regulate EMT. Hypoxia upregulates FGFR1, thereby increasing ERK phosphorylation, and upregulating ZEB1 to promote EMT and induce erlotinib resistance.^100^ Furthermore, HIF-2 targets the hypoxia-response element (HRE) of the Polo-like kinase 1 (Plk1) promoter. Plk1 induces EMT and leads to sunitinib resistance in ccRCC patients.^257^ Similarly, HIF-1α directly binds to the HRE of TWIST to regulate its expression and induce EMT.^258^ In addition, HIF can also induce overexpression of lncRNA-RP11-390F4.3 to regulate the expression of multiple EMT-TFs.^259^ Hypoxia can directly lead to EMT or expedite EMT by interacting with EMT-related pathways such as TGFβ, wnt, notch, and hedgehog through HIF.^260^

Some RTKs can also induce the expression of EMT-TFs through the ERK, JNK, and MAPK pathways. Some PTKs and their ligands, such as FGF/FGFR1, HGF/MET, IGF/IGFR, Gas6/AXL, and NGF/ TrkA have been implicated in EMT and TKI resistance.^170,261–264^ Among them, AXL has received more attention. AXL-mediated EMT is one of the resistance mechanisms of NSCLCs to EGFR-TKIs and ALK-TKIs, and HCCs to sorafenib.^265–267^

Since EMT is a reversible process, blocking EMT-related pathways in every stage can improve TKI resistance. Combination therapy with erlotinib and TGF-β type I receptor inhibitors effectively inhibited metastasis in erlotinib-resistant NSCLC cells.^268^ However, it is worth noting that dual inhibition of EGFR and TGFβR did not completely prevent acquired gefitinib resistance. Despite the reversal of EMT by TGFβR blockade, EGFR-resistant mutations like T790M became the predominant cause of the resistance after using TKI. These findings suggest that TKI resistance mechanisms may differ based on tumor type and stage of treatment.^254^ Replacement of EGFR-TKI is helpful to target the T790M mutation.

Targeting aberrantly activated AXL effectively attenuates TGF-β- and hypoxia-induced EMT.^269,270^ The AXL inhibitor, bemcentinib, and the MEK inhibitor, selumetinib specifically target mesenchymal and epithelial cells respectively, and their combination significantly inhibits the growth of tumors with both EMT and drug-resistant mutations. It successfully restores TKI sensitivity, making AXL a promising target for drug therapy.^271^ Since high expression of Aurora kinase family members is associated with EMT and drug resistance, inhibition of these proteins also restores drug sensitivity. TKI with alisertib, an AuroraA inhibitor, partly reverses the EMT and improves EGFR-TKIs resistance.^272^ Combining an AuroraB inhibitor with osimertinib can helpfully prevent EMT and improve TKI resistance. Mechanistically, the AuroraB inhibitor stabilizes BIM by reducing its ser87 phosphorylation and activates BIM-mediated mitotic catastrophe. Besides, it can transactivate the p53 upregulated modulator of apoptosis (PUMA), a molecule that binds and inhibits members of Bcl-2 anti-apoptotic protein and induces apoptosis through FOXO1/3.^253^

EMT is a critical biological process through which epithelial-origin malignant tumor cells acquire a high capacity for migration and invasion. EMT also allows tumor cells to escape apoptosis, degrade extracellular matrix, and resist TKIs. TME and EMT are closely related, as TME stimuli such as hypoxia and disordered extracellular signals cause abnormal intracellular EMT signals and induce EMT.

In fact, the underlying mechanism of EMT is markedly complex because of the following reasons. First, EMT inducers are various. It’s worth considering whether some factors dominate the EMT process compared to other factors, which can be called “determinant EMT inducers”. The predominant inducers have the potential to be drug targets. Second, EMT-related pathways are various, and interactions among which are even unclear. Third, epigenetic modifications can also regulate EMT and cause drug resistance. Fourth, EMT can also occur in normal cells, which needs to be distinguished from pathological EMT. Fifth, EMT is a dynamic and reversible process, and EMT-related TKI resistance may be a progressive step. It’s worthwhile to distinguish the cancer features in different EMT stages.

Discovering the molecular mechanisms of EMT and TKI resistance and exploring key molecules in EMT is crucial for understanding EMT-related TKI resistance.

#### Cellular metabolism

**Fig. 4:**
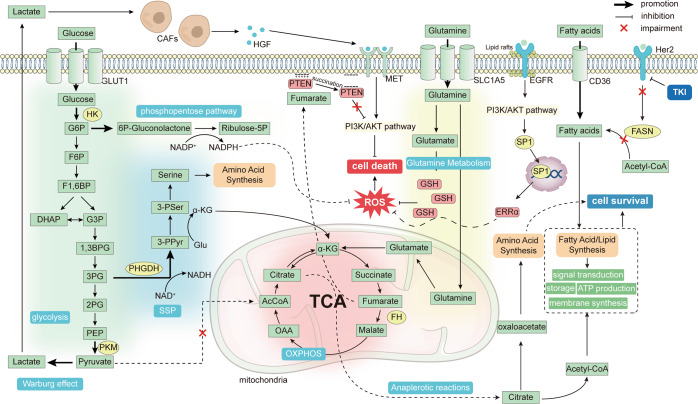
Impact of tumor metabolic reprogramming on TKI drug resistance. Under the pressure of TKI treatment, tumor cells undergo metabolic reprogramming to adapt to the altered environment caused by TKI, but the metabolic reprogramming of tumor cells is not the same in different contexts. Some drug-resistant cells mostly exhibit elevated activity of enzymes related to the glycolytic pathway to enhance glycolysis, and the Warburg effect also tends to be enhanced, leading to increased lactate production, which can stimulate CAFs to secrete HGF and activate MET on cancer cells. In addition, serine metabolism, the pentosephosphate pathway, and glutamine metabolism in tumor cells are also reprogrammed. The intermediates of these pathways can enter the tricarboxylic acid cycle to assist lipid and protein synthesis and promote cell survival, and some antioxidant substances, such as GSH and NADPH, produced midway can also reduce ROS levels to avoid cell death. The reprogramming of lipid metabolism can promote cell survival and resist cell death by affecting multiple processes, such as lipid composition of cell membranes, intracellular signaling, and sensitivity to oxidative stress, leading to TKI resistance

Tumorigenesis and tumor progression require metabolic reprogramming of tumor cells to meet the increased energy and nutrient demands and to reduce oxidative stress. And tumor cells compete with other cell types for nutrients and survival.^273^ Recent findings suggest the role of tumor metabolic reprogramming in TKI resistance induction and provide new insight into drug resistance improvement (Fig. 4).

A transcriptomic and proteomic analysis of imatinib-, nilotinib-, and dasatinib-resistant CML cell lines revealed their common metabolic profile. Compared to TKI-sensitive cells, which rely on mitochondrial oxidative phosphorylation (OXPHOS) for ATP production, the resistant cell lines significantly induced hypoxic signaling and had higher levels of the glycolytic pathway pentose phosphate pathway (PPP), and fatty acid metabolic pathway.^274^ Increased glycolysis was also observed in sunitinib-resistant RCC cell lines and EGFR-TKIs-resistant NSCLC cell lines.^275,276^ Increased glycolysis may be related to the upregulation of glycolysis-related enzymes and increased glucose uptake. Hexokinase (HK), the first glycolytic pathway enzyme, can be upregulated by hypoxia, which is associated with HCC resistance to regorafenib.^277^ Mechanistically, hypoxia stabilizes HIF-1α by ubiquitin-specific peptidase (USP29) and then transcriptionally activates HK.^278^ Pyruvate kinase M2 (PKM2), another rate-limiting enzyme of glycolysis, is overexpressed in TKI treatment, accompanied by high levels of lactate production. PKM2 overexpression is associated with imatinib resistance in CML cell lines and gefitinib resistance in colorectal cancer.^279,280^ In addition to inducing lactate production, PKM2 can directly activate the PI3K/AKT pathway and STAT3-related pathway to develop drug resistance. TKIs can induce IL-6 release, thereby upregulating glucose transporter type 1 (GLUT1) and increasing glucose uptake for proliferation. In addition, IL-6 can further increase its own levels by activating the NF-κB pathway and forming a positive feedback loop.^276^

Increased levels of glycolysis are accompanied by a shift from the pyruvate-mediated tricarboxylic acid (TCA) cycle to Warburg metabolism with lactate production increase.^274^ Increased lactate levels lead to the upregulation of lactate transporter proteins, glycolytic enzymes, and negative regulators of the TCA cycle in CAFs. High lactate levels induce CAFs to overexpress HGF in an NF-κB-dependent manner, which binds to and activates MET on cancer cells, and leads to gefitinib resistance in NSCLC. Therefore, inhibition of glycolysis may restore drug resistance.^275^ Higher lactate concentrations are also accompanied by upregulation of carbonic anhydrase 1 (CA1) and α-synuclein (α-Syn) in some cell lines.^274^ Some studies suggest that CA1 may be involved in fatty acid de novo synthesis,^281^ while α-Syn alters the metabolite into mitochondrial flux to attenuate oxidative stress and apoptosis.^282^ All of which are the manifestation of metabolic reprogramming.

However, it should be noted that elevated levels of glycolysis and downregulated levels of OXPHOS may not apply to all TKI-resistant cells. Metabolic profiling of erlotinib-resistant pancreatic ductal adenocarcinoma (PDAC) cells showed decreased levels of glycolysis and increased levels of PPP enzyme. PPP increases the NADPH/NADP^+^ ratio to protect cells against reactive oxygen species (ROS).^283^ Another study on EGFR-TKI-resistant NSCLC cell lines showed that resistant cell lines reduce glucose uptake and glycolytic capacity but enhance mitochondrial OXPHOS function. Inhibition of mitochondrial respiration by metformin, which targets OXPHOX complex I, may inhibit cell proliferation.^284^ Metabolic changes in different drug-resistant cancers are not all the same. Thus, individualized metabolic profiling is necessary.

Metabolomic analysis of sunitinib-resistant RCC cell lines showed a significant increase in glutamine transporter protein (SLC1A5) expression apart from the same increase in glycolytic enzymes and lactate.^285^ In addition, ROS1-TKI crizotinib-resistant NSCLC cell lines also showed increased glutamine metabolism and fatty acid synthesis dependence.^286^ High levels of intracellular glutamine can be converted into glutamate and then α-ketoglutarate (αKG) into TCA, which not only meets the increased energy demand of tumors but also helps the synthesis of amino acids and lipids through the anaplerotic reaction. Furthermore, glutamine is a precursor of glutathione (GSH), which is a powerful antioxidant that inhibits oxidative stress-induced cell death. Tumor cells initially rely on glutamine metabolism for energy, and the reliance situation even becomes severe in the development of resistance, and its role in antioxidant defense is more important in drug resistance. However, the role of glutamine metabolism in tumor biology goes beyond the two aforementioned aspects,^287^ which has the potential to reverse TKI resistance.

Lipid metabolic reprogramming can also confer tumor TKI resistance, among which lipid peroxidation is closely related. Fatty acids are classified into saturated fatty acids, monounsaturated fatty acids (MUFAs), and polyunsaturated fatty acids (PUFAs). Normal cells tend to meet fatty acids demands through exogenous uptake, while tumor cells rely on the de novo synthesis of fatty acids to meet their needs. Tumor cells can also increase membrane lipid saturation to reduce the sensitivity to oxidative stress and resist cell death.^288^ Sterol regulatory element-binding protein 1 (SREBP1) is responsible for the de novo synthesis, whose high expression can be seen in gefitinib-resistant cells. Inhibition of SREBP1 elevates ROS and lipid peroxidation levels, suggesting that SREBP1-induced de novo synthesis of fatty acids can prevent cell death.^289^ However, in HER2-TKI lapatinib-resistant breast cancer cell lines, lapatinib upregulated CD36 to increase exogenous fatty acid uptake instead of fatty acid de novo synthesis. Mechanistically, fatty acid synthase (FAS), which is responsible for de novo synthesis, can interact with HER2, so HER2 blockade inhibits FAS and fatty acid de novo synthesis. However, tumor cells can develop a compensatory mechanism to maintain fatty acid supply through exogenous fatty acid uptake, which is also a manifestation of metabolic reprogramming.^290^

Cholesterol metabolism is also one of the lipid metabolism pathways. Studies have shown that gefitinib downregulates the expression of peroxisome proliferator-activated receptor alpha (PPARα), LXRα, and ABCA1, leading to dysregulation of cholesterol efflux and induction of drug resistance in NSCLC cell lines.^283^ The exact relationship between cholesterol accumulation in cancer cells and drug resistance is unclear, but the combination of fenofibrate, a lipid-lowering drug that can upregulate the PPARα expression, and gefitinib can promote apoptosis in resistant cells. Activation of the PPARα/AMPK/AKT/FoxO1 pathway was involved in this effect. FoxO1 regulates the expression of several molecules involved in the cellular oxidative stress response, suggesting a role for PPARα in lipid peroxidation.^291^ Another study on osimertinib-resistant NSCLC cell lines revealed a possible role of cholesterol in drug resistance. Cholesterol accumulation in lipid rafts promotes EGFR-Src interaction, activates ERK/SP1 signaling via Src and enhances estrogen-related receptor alpha (ERRα) expression, which regulates ROS levels and maintains cell proliferation, leading to drug resistance.^292^ Phospholipid metabolism is also associated with drug resistance. An elevated level of lysophosphatidylcholine acyltransferase 1 (LPCAT1), an important enzyme of phospholipid metabolism, was observed in LUAD-resistant cell lines. LPCAT1 induced lysophosphatidylcholine to convert to a saturated state from an unsaturated one, remodeling the cell membrane lipids’ constitution. Cell membrane lipids remodeling led to activation of the EGFR/PI3K/AKT pathway, expedited cell proliferation and LPCAT1 expression, forming a positive feedback.^293,294^

There are also several amino acid metabolic pathways associated with drug resistance. First, phosphoglycerate dehydrogenase (PHGDH), a key enzyme of the serine synthesis pathway (SSP), is a major driver of sorafenib resistance in HCC. Inhibition of PHGDH can inhibit SSP, reduce the production of αKG, serine, and NADPH II, and induce cell death by increasing ROS production. Sorafenib induces PHGDH expression,^295^ leading to tumor cell survival and drug resistance. Second, methionine metabolism, or the whole sulfur amino acid (SAA) metabolism, which is involved in protein synthesis, sulfur metabolism, redox homeostasis, epigenetic modification, and signal transduction. Methionine metabolism is also associated with sorafenib resistance in HCC. Hepatocyte nuclear factor 4 alpha (HNF4α) is a regulator of SAA and is negatively correlated with EMT in HCC. Inhibition of SAA metabolism by downregulating HNF4α enhanced EMT, and exacerbated sorafenib resistance, with an unknown mechanism.^296^ Since methionine is an essential amino acid and its bioavailability relies on diet, regulating methionine intake may be beneficial for tumor treatment and reversal of drug resistance.^297^

The metabolites also have non-metabolic modifying functions. Fumarate accumulates in cells lacking fumarate hydratase (FH) and directly reacts with PTEN at cysteine-211 (C211) to form S-(2-succino)-cysteine. Succinated C211 blocks the binding of PTEN to the cell membrane, thereby reducing its inhibitory effect on the PI3K/AKT pathway, leading to sustained activation of the pathway and further sunitinib resistance of type 2 papillary renal cell carcinoma.^298^ Simultaneous use of AKT inhibitors or enhancing FH expression may improve drug resistance.

Cancer cells gradually develop metabolic adaptations and resistance during TKI treatment, suggesting that tumor metabolic reprogramming is an effective mechanism for tumor cells to resist TKI. Changes in metabolite levels in the TME offer new insights into TKI resistance. There are studies supporting the use of glycolysis inhibitors to ameliorate sunitinib resistance.^299^ Thus, targeting tumor metabolic adaptation and altering metabolites levels and composition of TME may improve cancer treatment.^300^ In addition, metabolic changes help diagnose tumors and detect TKI resistance on a macroscopic scale. In other words, identifying specific metabolites or combinations of several metabolites may be helpful to characterize an individual tumor, in which metabolomics has great potential.^301^ Metabolomics was used to characterize the metabolic profile of cancer cachexia. A diagnostic model using serum myostatin, leucine, and phenyl acetate levels can reach a diagnostic accuracy of 94.64%, showing potential for practical application.^302^ It is worth exploring its application in TKIs resistance to tumors.

### Cell death inhibition

Cell death resistance is a hallmark of cancer. Cell death pathways include apoptosis and non-apoptotic forms, such as ferroptosis, autophagy, necroptosis, and pyroptosis. Tumor cells that develop death resistance can survive and proliferate continuously, resulting in tumor survival, recurrence, and treatment resistance.

#### Apoptosis inhibition

In cancer, dysregulated apoptotic signaling, particularly the activation of the anti-apoptotic pathways, allows cancer cells to escape apoptosis, leading to uncontrolled proliferation and TKI resistance.

The Bcl-2 family plays a vital role in regulating apoptosis and controls the intrinsic mitochondrial apoptosis pathway. The family can be divided into two major groups: one group exerts anti-apoptotic effects, such as bcl2 apoptosis regulator (BCL-2), BCL-X, BCL-W, and MCL1 apoptosis regulator (MCL-1), and the other one plays a pro-apoptotic role, such as BIM, PUMA, and NADPH oxidase activator (NOXA).^303^ Family members antagonize each other and regulate cell death. Overexpression of anti-apoptotic members or low expression of pro-apoptotic members can lead to survival and drug resistance.^115^

Studies have shown that BIM knockdown can significantly attenuate or eliminate the therapeutic effects of third-generation EGFR-TKIs, indicating that low BIM expression is a major cause of TKI resistance. BIM downregulation occurs during the overactivity of PI3K/AKT and RAS/MAPK. These pathways phosphorylate the transcription factor FOXO and downregulate BIM expression.^304^ For example, hypoxia upregulates FGFR to activate the RAS/MAPK pathway, thereby downregulating BIM expression and inducing osimertinib resistance.^100^ EMT-TFS can also downregulate BIM. In addition, epigenetic modification can also change BIM expression. BIM deletion polymorphisms are one of the mechanisms of primary resistance to EGFR-TKIs in approximately 21% of the East Asian population.^305^ They express BIM molecules without pro-apoptotic activity, resulting in impaired apoptosis. NSCLC with BIM deletion polymorphism was resistant to all three generations of EGFR-TKIs. But it is still controversial whether BIM deletion polymorphism affects the survival of patients, and further studies are needed.^35^

Apart from BIM, some cell lines also overexpress anti-apoptotic proteins such as BCL-2. Similar to BIM, high BCL-2 expression occurs because of PI3K/AKT and RAS/MAPK pathways overactivity.^306^ Epigenetic modifications can also regulate BCL-2 expression. Elongator (ELP) complexes can promote MCL-1 expression through tRNA modification, resulting in triple-negative breast cancer resistance to erlotinib.^307^ Other mechanisms, such as gene hypomethylation, gene rearrangements, and silencing mutations in noncoding RNAs that regulate BCL-2, can also upregulate BCL-2.^303^ However, some of these factors have not yet been demonstrated to be associated with TKI resistance. BCL-2 leads to TKI resistance partly by downregulating BIM through binding to contemporaneous low expression of BIM.

The induction of apoptosis is a potential solution for drug resistance. Upregulation of pro-apoptotic factors or downregulation of anti-apoptotic factors is effective. The combination of vorinostat, an inducer of BIM expression, and metformin, an inhibitor of anti-apoptotic proteins, enhanced the sensitivity of drug-resistant NSCLC to gefitinib.^308^ Inhibition of Mcl-1 and activation of Bax improve acquired resistance to osimertinib and other third-generation EGFR-TKIs in NSCLC.^309^ Similarly, inducing the expression of NOXA and bound Mcl-1 by ixazomib, a proteasome inhibitor, attenuated resistance to the ALK-TKI alectinib in ALK-rearranged NSCLC cell lines.^310^ The MCL-1 inhibitor, S63845, reversed drug resistance by enhancing apoptosis in regorafenib-resistant CRC cells.^311^ In addition, inhibition of DNA damage-induced apoptosis suppressor (DDIAS), an anti-apoptotic protein that binds to and promotes STAT3 signaling to inhibit apoptosis, led to apoptosis in gefitinib-resistant cells.^312^ In cancer cells with high expression of anti-apoptotic molecules, simply elevating the expression of pro-apoptotic molecules may not be sufficient to induce apoptosis. Hence, it may be more reasonable to directly target anti-apoptotic proteins instead of upregulating pro-apoptotic proteins.^310^ Mitochondria are the junction of apoptosis, and interference with mitochondrial function can induce apoptosis. Piperlongumine, the piper longum extract, can strongly induce ROS production and mitochondrial dysfunction and activate the AMP-activated protein kinase (AMPK) pathway, inhibiting cell growth and survival.^313^ Blocking the cell cycle under certain conditions can also induce apoptosis. Cyclin-dependent kinase (CDK) is the key kinase in cell cycle regulation, and CDK inhibitors can attenuate TKI resistance. TKI in combination with CDK7/12 inhibitor THZ1, CDK9 inhibitors alvocidib, and dinaciclib had a greater impact on apoptosis in drug-resistant cells.^314–316^

#### Non-apoptotic cell death inhibition

In addition to apoptosis, there are several forms of programmed cell death, such as ferroptosis, autophagy, necroptosis, and pyroptosis. These non-apoptotic forms of cell death are also closely related to TKI resistance.

##### Ferroptosis

Ferroptosis is a form of programmed cell death caused by iron-dependent peroxidation of polyunsaturated phospholipids in the cell membrane, and it is activated by two main pathways.^317^ The extrinsic pathway refers to the inhibition of cell membranes transport proteins such as cysteine/glutamate transport proteins, also known as the xc- system, or the activation of ferroportin. The former reduces GSH levels by decreasing GSH precursor uptake and increasing peroxides accumulation, while the latter increases intracellular iron and causes PUFA peroxidation by Fenton’s reaction with intracellular ROS. The intrinsic pathway refers to the direct inhibition of intracellular antioxidant enzymes such as glutathione peroxidase 4 (GPX4), leading to peroxides accumulation.^318^ The accumulation of oxidants in both pathways finally leads to ferroptosis. Tumor cells develop several mechanisms to escape ferroptosis-induced cell death, causing drug resistance. Inhibition of lipid peroxidation prevents ferroptosis and enhances cell survival and drug resistance.

Nuclear factor erythroid 2-related factor 2 (NRF2) pathway activation prevents ferroptosis and increases TKI resistance. Sorafenib induces p62 expression to block NRF2 ubiquitinated degradation and to enhance its nuclear translocation by inactivating Kelch-like ECH-associated protein 1 (KEAP1).^319^ Sirtuin 6 (SIRT6) and inhibitor of apoptosis-stimulating protein of p53 (iASPP) also bind to Keap1 to strengthen NRF2-mediated antioxidant defense. Overexpression of SIRT6 is associated with sorafenib resistance in gastric cancer cell lines, but iASPP need further exploration.^320,321^ In the nuclear, NRF2 binds to the cis-transcriptional regulatory element antioxidant response element (ARE). It interacts with transcriptional coactivators such as MAF-BZIP transcription factor G (MAFG), activating the transcription of NAD(P)H quinone dehydrogenase 1 (NQO1), heme oxygenase 1 (HMOX1), and ferritin heavy chain 1 (FTH1) to increase the expression of antioxidants and inhibit ferroptosis. Metallothionein-1G (MT-1G), ABCC5, GPX4, and superoxide dismutase (SOD) are also transcriptional targets of NRF2. MT-1G is a ROS scavenger that negatively regulates ferroptosis.^320,322–324^ ABCC5 stabilizes SCL7A11 to increase GSH precursor uptake and simultaneously enhance sorafenib efflux, leading to TKI resistance.^325^ Apart from NRF2 pathways, the Hippo pathway downstream transcriptional complex YAP/TAZ (Tafazzin)-TEAD (TEA domain transcription factor) is a key factor for sorafenib resistance in HCC. As a transcription factor, YAP/TAZ directly induces xc^-^system subunit SLC7A11 expression and synergistically induces its expression by increasing activating transcription factor 4 (ATF4) stability. High SLC7A11 expression increases intracellular GSH synthesis, attenuates lipid peroxidation, and inhibits ferroptosis.^326^ Additionally, Tumor cells-derived stearoyl-CoA desaturase-1 (SCD1), tumor endothelial cells (TECs)- and adipocytes-derived fatty acid-binding protein 4 (FABP4) induce ferroptosis resistance in breast cancer cells treated with sunitinib or sorafenib.^327^ Mechanistically, SCD1 desaturates fatty acids to generate MUFAs, while FABP4 maintains lipid droplet (LD) formation in tumor cells to reduce LD destruction-induced ROS production and inhibit the ferroptosis.^328^ Moreover, Sorafenib initially induces ferroptosis inhibiting cysteine uptake, but intracellular cysteine deficiency may lead to mitochondrial membrane potential hyperpolarization and mitochondrial dysfunction,^329^ further activating PI3K-RAC1(RAC family small GTPase1)-PAK1 signaling and enhancing micropinocytosis to replenish intracellular depleted cysteine and resist ferroptosis.^330^

GPX4 overexpression is observed in the hyper mesenchymal state caused by EMT and is associated with erlotinib resistance.^331^ GPX4 can scavenge peroxides, allowing cells to resist ferroptosis, but the relationship between EMT and GPX4 is not clear, and EMT can also lead to drug resistance through non-ferroptosis mechanisms. Consequently, the relationship between EMT and ferroptosis deserves further studies. The current study shows that GPX4 inhibition strongly induces ferroptosis of mesenchymal-like cancer cells compared with its negligible effect on epithelioid cancer cells, leading to the speculation that highly mesenchymal-like cells are more susceptible to ferroptosis, which may be related to high baseline transcription of ZEB1 in mesenchymal-like cells. ZEB1 induces the upregulation of PPARγ, the primary regulator of hepatic lipid metabolism, to promote lipid oxidative metabolism.^332^ In addition, ZEB1 induces plasma membrane remodeling via EMT, which is the site of lipoxygenase-induced PUFA oxidation. Therefore, it may affect peroxide accumulation, but more specific mechanisms remain to be investigated.

Inducing ferroptosis can significantly decrease drug-resistant tumor cell viability.^317^ We have reviewed that TKI resistance is linked to ferroptosis inhibition. Therefore, re-activating ferroptosis may reduce the TKI resistance. Inhibition of GPX4 is an effective method to target the intrinsic pathway.^333^ Inhibition of GPX4 could overcome the EGFR-TKI resistance by inducing ferroptosis in NSCLC and LUAD cell lines.^331,334^ Reducing GSH precursor uptake, increasing intracellular ferric ion concentration, and increasing ROS levels are helpful for extrinsic pathways. Amiloride inhibited the effect of macropinocytosis, and the combination with sorafenib decreased HCC cells’ drug resistance.^330^ The combination of the lysosomal disruptors, Siramesine, and lapatinib upregulated transferrin to increase ferric ion transport into the cancer cells and downregulated ferroportin-1 to decrease ferric ion transport out of the cell, thereby inducing intracellular ferric ion accumulation, ROS production, and ferroptosis.^335^ Moreover, disulfiram-copper (DSF/Cu) significantly damaged mitochondria, accelerated ROS accumulation, induced lipid peroxidation, and potentiated the cytotoxic effect of sorafenib in vitro and vivo by simultaneously inhibiting the signal pathway of NRF2.^336^ Additionally, cryptotanshinone (CTS) may inhibit the expression of the antioxidants such as catalase (CAT), HMOX1, and SCD and alleviate gefitinib resistance in NSCLC cells.^294^ Since lipid peroxidation happens throughout ferroptosis, targeting lipid metabolism to induce ferroptosis or, even more broadly, targeting the tumor metabolic reprogramming may improve drug resistance. For instance, there are certain crosstalks between mannose metabolism and lipid metabolism. Inhibition of high mannose phosphonic isomerase (MPI) can decrease TLT3-TKI resistance in AML with FLT-ITD. Mechanistically, MPI knockdown triggers defective protein glycosylation, which can induce the unfolded protein response (UPR) and drive ATF6 transcriptionally inhibits PPARα, increasing PUFA levels and inducing ferroptosis.^337^

In summary, some TKIs can induce ferroptosis, allowing tumor cells to develop drug resistance. During TKI administration, tumor cells can resist lipid peroxidation-induced ferroptosis by activating antioxidant pathways, enhancing GSH biosynthesis, and upregulating GPX4, leading to drug resistance. In addition, lipid metabolism reprogramming is closely related to ferroptosis, and they can potentiate each other, increasing tumor cells survival. Moreover, numerous factors can influence ferroptosis, such as P53 mutations and hypoxia, and ferroptosis may cause inflammation-related immunosuppression in TME, enhancing tumor growth.^318^ Therefore, targeting ferroptosis is rational to reverse TKI resistance.

##### Autophagy

Autophagy is both a tumor suppressor pathway and a survival mechanism for tumor cells to withstand metabolic stress and resist treatment.^337^ mTOR is a critical regulator of autophagy. TKIs can induce autophagy by reducing mTOR^338^ phosphorylation through several pathways such as PI3K/AKT, MAPK, and JAK/STAT.^339^ There are studies discussing the detrimental role of TKIs-induced autophagy.^340–342^ However, given the paradoxical role of autophagy, the outcome of TKI-induced autophagy is not definitively determined yet. Here, autophagy-mediated TKI resistance was reviewed.

In FLT-TKIs resistant AML cells with FLT3-ITD, FLT3-ITD-mediated aberrant activation of FLT3 induced autophagy by upregulating the activity of ATF4. High-level autophagy results in cancer cell survival and resistance to FLT-TKIs.^343^ In erlotinib-resistant NSCLC, high GATA binding protein 6 (GATA6) and a threefold increase in autophagic activity were observed. In contrast, GATA6 knockdown reduced autophagy and restore drug sensitivity, suggesting the interaction among GATA6, autophagy, and TKI resistance.^344^ Furthermore, abnormal AXL activation also increased autophagic flux in NSCLC cell lines. It prevented caspase-mediated apoptosis, leading to resistance to the first- and third-generation EGFR-TKIs erlotinib and rociletinib. The cytoprotective effect leading to drug resistance was independent of AXL-induced EMT.^345^ Apart from mTOR-induced autophagy, AMPK could induce autophagy in response to intracellular ATP deficiency caused by nutrient deprivation or mitochondrial function inhibition. It was shown that BCR-ABL TKI nilotinib induces AMPK activation and phosphorylates Unc-51-like autophagy activating kinase 1 (ULK1). ULK1 activates downstream targets such as autophagy-related 13 (ATG13), thereby increasing autophagic flux to maintain CML cell survival. This shows that TKI may alter tumor energy or metabolic status and adaptively activate autophagy to maintain cell survival.^346^ Mitochondrial autophagy is a specific type that can be activated by PTEN-induced kinase 1 (PINK1)-Parkin signaling. Hypoxia/HIF-1α can upregulate miR-210-5p in HCC, downregulating ATPase family AAA domain-containing 3 A (ATAD3A) expression and triggers mitochondrial autophagy via the PINK/Parkin pathway and leads to sorafenib resistance. The mechanism may be overactivated mitochondrial autophagy and reduced ROS production, leading to death resistance.^347^

In summary, autophagy can support tumor cell proliferation and contribute to drug resistance in some cases because of its role in energy and nutrient metabolism. However, the detailed mechanism of autophagy-dependent proliferation and apoptosis resistance needs further investigation. In autophagy-induced drug resistance, inhibition of autophagy can restore TKI sensitivity. Afatinib combined with the autophagy inhibitor, CQ, has greater anti-cancer efficacy.^341^ Azithromycin combined with gefitinib enhances pancreatic cancer cell death by inhibiting autophagy.^348^ Furthermore, a phase II randomized clinical trial (CHOICES) demonstrated that imatinib combined with HCQ for chronic phase CML achieved a higher (20.8%, P = 0.059) cytogenetic remission rate at 24 months compared with imatinib alone.^349^

Paradoxically, some studies have shown that combining TKIs and autophagy inhibitors such as Beclin-1 tyrosine phosphomimetic mutant or BCL-2 enhances tumor growth, dedifferentiation, and TKI resistance.^350^ These observations suggest that autophagy inhibition can lead to TKI resistance, but the exact mechanism is unclear. Beclin1-induced autophagy requires Beclin autophosphorylation to be released from BCL2/BCL-XL proteins, showing the link between apoptosis and autophagy.^351^ The relationship between apoptosis and autophagy may contribute to the development of drug resistance. Moreover, hypercholesterolemia is associated with sorafenib resistance. High SCAP induced by disordered lipid metabolism can inhibit autophagy by downregulating AMPK activity.^351^ However, the effect of decreased autophagic flux on drug resistance is not clear.^337^

In cases where autophagy inhibition is harmful, the induction of autophagy is beneficial for TKI therapy.^352^ Serine and arginine-rich splicing factor 1 (SRSF1) is an autophagy suppressor, and its knockdown may activate autophagy and inhibit the proliferation of gefitinib-resistant cancer cells.^353^ Cannabidiol promotes mitochondrial autophagy in CML cells by activating transient receptor potential vanilloid type 2 (TRPV2). Cannabidiol exerts a synergistic effect with imatinib in imatinib-resistant CML cells.^354^ In AML carrying FLT3-ITD, FLT-3-ITD molecules were detected in the autophagosome after induction of autophagy with the proteasome inhibitor bortezomib. It suggests that autophagy is involved in the posttranslational degradation of aberrant PTKs, and induction of autophagy is undoubtedly beneficial in this case. Bortezomib can sensitize cancer cells to FLT-3 TKI quizartinib in combination therapy.^355^

More interestingly, lapatinib, a dual inhibitor of EGFR and ERBB2, promotes autophagy in cells expressing only EGFR but inhibits autophagy in cells expressing ERBB2.^356^ These findings suggest that TKIs may act differently in tumors with different mutation profiles. The contradictory results regarding the role of autophagy in TKI resistance reflect the uncertain role of autophagy in cancer treatment.^357^ Therefore, some concerns require further exploration: (I) the relationship between abnormal PTK activation and autophagy; (II) whether all TKIs induce autophagy and how to define the protective and toxic effects of TKI-induced autophagy on cancer cells. Since autophagy is associated with both apoptotic cell death and some types of non-apoptotic cell death, exploring the intrinsic link between different types of cell death is pivotal for understanding TKI resistance.

##### Other nonapoptotic cell death inhibition

Necroptosis and pyroptosis are also emerging forms of nonapoptotic cell death, and recent findings suggest their involvement in TKI resistance.

Necroptosis, also called programmed necrosis, is a necrotic cell death mediated by RIPK1 and RIPK3 kinase. Under the stimulation of TNFα, lipopolysaccharide/Toll-like receptors, interferon (IFN), T-cell receptors, etc.,^358^ cells form a necrosome complex (RIPK1, RIPK3, MLKL), oligomerizing and translocating mixed lineage kinase domain-like pseudokinase (MLKL) to the cell membrane to initiate necroptosis. It causes the release of damage-associated molecular patterns (DAMPs) and inflammation.^359^ Some TKIs induce necroptosis, so it is reasonable to consider the role of necroptosis inhibition in TKI resistance.

Hypoxia is also important. A TKI with anti-angiogenesis effects exacerbates hypoxia. HIF induces HSP90α expression, which binds the necrosome complex, induces MLKL degradation by autophagic lysosomes, blocks necroptosis, and induces HCC resistance to sorafenib.^360^ Epigenetics contributes to necroptosis resistance. It was shown that overexpression of miR-92a-1-5p in Ph+CML cells inhibits MLKL expression and correlates with BCR-ABL-TKI resistance.^361^ ROS also induces necroptosis. It can promote RIP3 autophosphorylation and necrosome complex assembly.^362^ These findings raise the question of whether reduced ROS production prevents necroptosis during treatment with TKIs.

Interestingly, sorafenib was shown to directly target RIPK1 and RIPK3 to block the second mitochondria-derived activator of caspases (Smac)-induced necroptosis in CML cell lines. It is consistent with clinical reports that low micromolar concentrations of sorafenib could inhibit necroptosis.^363^ An animal study explained that sorafenib acts differently depending on the concentration.^364^ Lower concentrations inhibit necroptosis without affecting NF-κB activation, while higher concentrations induce non-necroptotic forms of cell death. In addition to sorafenib, ponatinib and pazopanib have also been identified as inhibitors of necroptosis.^365^ In other words, some TKIs initially inhibit necroptosis, but the pros and cons of this effect are unknown, and further studies regarding the relationship between TKI and necroptosis are needed.

Targeting necroptosis-associated molecules is a promising approach to improving drug resistance. TNF-α is a trigger molecule for necroptosis, but upregulation of its expression is ineffective in inducing necroptosis, and TNF-α levels exactly positively correlate with sorafenib resistance, for high TNF-α will lead to EMT and it can also induce activation of the NF-κB pathway to avoid cell death.^366^17-demethoxygeldanamycin (17-AAG) is a targeted inhibitor of HSP90α. Animal studies have shown that 17-AAG reversed sorafenib resistance in HCC.^360^ Moreover, shikonin is a small molecule from traditional Chinese medicine that can induce necroptosis. It activated the necrosome complex and overcame the resistance of Ph^+^ CML to BCR-ABL TKI and RCC cells to sunitinib.^361,367^ In addition, shikonin downregulated cell cycle-activating proteins and inhibited the proliferation of drug-resistant cells through cycle arrest. It also directly inhibited the AKT/mTOR pathway and enhanced TKI function.^367^ Apurinic/pyrimidinic endonuclease 1 (APE1) is responsible for DNA damage repair. APE1 inhibitors induced DNA damage in NSCLC cell lines, thereby inducing apoptosis, pyroptosis, and necroptosis and overcoming erlotinib resistance, suggesting that inducing cell death by targeting DNA repair can improve cancer treatment.^368^ Nuclear protein 1 (NUPR1) is not expressed in normal tissues but is highly expressed in precancerous lesions of the liver. Inhibition of its expression by ZZW-115 resulted in a substantial decrease in intracellular ATP concentrations and induced apoptosis and necroptosis, suggesting that NURP1 is an effective drug target.^369^

Pyroptosis is an intensely inflammatory lytic programmed cell death. The typical pyroptosis pathway is initiated by assembling large supramolecular complexes called inflammasomes in response to signaling stimuli.^370^ Inflammasomes can activate specific caspases, which cleave and activate the effector molecule gasdermin (GSDM). It causes pore formation on the cell membrane, leading to cancer cells swelling and rupture,^371^ resulting in the release of proinflammatory molecules and inflammatory cells infiltration.

The relationship between pyroptosis and cancer is now quite unclear, the pyroptosis-mediated inflammatory response can induce an immune response that contributes to tumor regression, but pyroptosis may sometimes induce a supportive TME.^372^ Theoretically, pyroptosis inhibition may help TKI resistance if the TKI acts through pyroptosis. However, only a few TKIs have been shown to kill tumor cells through pyroptosis. Studies have shown that sorafenib can induce pyroptosis in TAMs and release inflammatory mediators to induce NK cells-mediated cytotoxicity.^373^ Dasatinib can directly induce pyroptosis in tumor cells.^374^ Pyroptosis inhibition may help overcome drug resistance, but it has been poorly explored. In general, a few studies report necroptosis and pyroptosis with TKI resistance, and in-depth studies of both can help find new therapeutic targets.

**Fig. 5:**
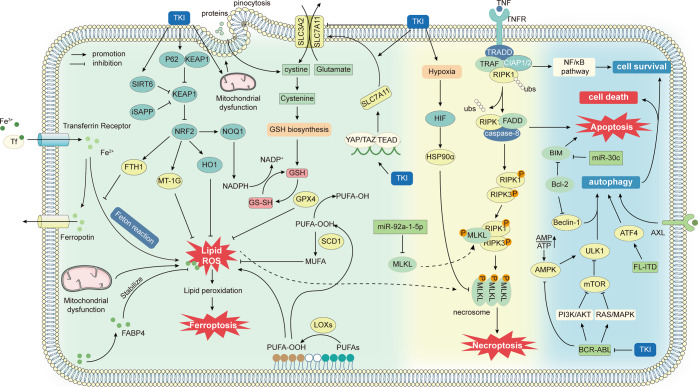
Cell death inhibition in TKI resistance. Under TKI pressure, tumor cells can resist apoptosis by downregulating proapoptotic molecules and/or upregulating apoptosis-inhibiting molecules. They can also resist ferroptosis by reducing lipid peroxidation through adaptive activation of antioxidant pathways and enhanced production of antioxidant substances such as GSH. Autophagy can also affect TKI resistance, but the relationship between autophagy levels and TKI resistance varies between contexts. Necroptosis is initiated by the necrosome complex, and factors such as ncRNA and hypoxia can inhibit necroptosis by suppressing the assembly of the necrosome complex

Death resistance is the characteristic of cancer cells. The adaptive changes that occur in the TME during TKI treatment can exacerbate death resistance and lead to the development of drug resistance. However, programmed cell death is a double-edged sword, and inducing cancer cell death may not be totally beneficial for cancer treatment.^375^ Selective manipulation of cell death is a potential approach for tumor eradication and reversal of drug resistance (Fig. 5).

### Epigenetic modification

Epigenetics bridges the gap between genotype and phenotype. It regulates chromatin structure and gene expression by covalent modification of histones and nucleic acids through multiple modalities without altering nucleotide sequences.^376^ Epigenetics is closely associated with carcinogenesis and drug resistance and is involved in drug-tolerant persisters (DTPs).^377–379^

#### Noncoding RNA (ncRNA)

ncRNAs do not encode proteins but are involved in posttranscriptional modifications. ncRNAs mainly include microRNAs (miRNAs), long noncoding RNAs (lncRNAs), and circular RNAs (circRNAs). ncRNAs regulate gene expression without altering gene sequence and are deeply involved in epigenetic modification. Their expression levels are often abnormal in tumors, which is associated with tumor progression and TKI resistance (Table 3).

**Table 3:** ncRNAs related to TKI resistance

ncRNAs	Upstream molecules or pathways	Target molecules or pathways	Effect
miRNA			
miR-212^455^(↓)	—	ABCG2	Increased drug efflux
miR-452-5p^135^(↑)	—	DUSP6	Activating the RAS/MAPK pathway
miR-210-5p^347^(↑)	Hypoxia/HIF	ATAD3A/ PINK1/PARKIN	Activation of mitochondrial autophagy
miR-92a-1-5p^361^(↑)	—	MLKL	Necroptosis inhibition
miR-200^247^	—	ZEB1/2	EMT
miR-186^247^	—	TWIST	EMT
miR-34a^247^	—	SNAIL	EMT
miRNA-125-5p^382^(↑)	—	ataxin-1	EMT
miR-424^383^(↑)	—	CBX4	nuclear translocation of YAP1 cancer stem cell phenotype
miR-25-3p^384^(↑)	—	PTEN	Activating the PI3K / Akt pathway to induce M2 polarization of macrophages
miR-130b-3p^384^(↑)	—		
miR-125d-5p^385^(↑)		c-Jun/PUMA	Apoptosis resistance
miR-494^386^(↑)	—	PTEN, P27, PUMA	Activating the PI3K / Akt pathway Apoptosis resistance
miR-30a-5p^387^(↓)	—	CLCF1	Activating the PI3K / Akt pathway Promoting glycolysis
miR-30b/c^388^(↑)	MET	BIM	Apoptosis resistance
miR-335^389^(↑)	—	AXL	Activating the PI3K / Akt pathway
miR-196a^390^(↑)	NRF2	GLTP	Cell proliferation
miR-221^392^(↑)	NF-κB pathway	p27^kip1^	Apoptosis resistance
lncRNA			
lncRNA-RP11-390F4.3^259^(↑)	hypoxia/HIF-1α	Snail,Twist1, ZEB1, ZEB2	EMT
lncRNA-ECVSR^394^(↑)	—	ERβ/HIF2-α	cancer stem cell phenotype increasing vascular mimicry
lncRNA-CASC9^395^(↑)	—	EZH2/DUSP1	Activating the RAS/MAPK pathway
LncRNA-PCAT-1^396^(↑)	—	PI3K/Akt pathway	Activating the PI3K / Akt pathway
LncRNA-HIF1A-AS2^397^(↑)	HIF-1α	miR-146b-5p/IL-6/STAT3	Activating the IL-6/STAT3 pathway to promote proliferation
lncRNA-RP11616M22.7^398^(↑)	—	RASSF	Regulation of hippo pathway
circRNA			
circFN1^399^(↑)	—	miR-1205/E2F1	Cell proliferation and apoptosis resistance
circFOXM1^400^(↑)	—	miR-1324/MECP2	Cell proliferation and apoptosis resistance
circRNA hsa_circ_0005576^401^(↑)	—	miR-512-5p/IGF-1R	Alternative activation of PTK
circRNA-SORE^402^(↑)	—	YBX1	apoptosis resistance
circ-E-Cad^405,406^(↑)		C-E-Cad/EGFR	Alternative activation of PTK
CircRNA-ASK1^426^(↓)	YTHDF2	ASK1-272a.a/PI3K/AKT	Activating the RAS/MAPK pathway

*ABCG2* ATP Binding Cassette Subfamily G Member 2, *AKT* AKT serine/threonine kinase, *ATAD3A* ATPase family AAA domain containing 3a, *AXL* AXL receptor tyrosine kinase, *BIM* bcl-2 interacting mediator of cell death, *CBX4* chromobox 4, *CLCF1* cardiotrophin-like cytokine factor 1, *DUSP* dual specificity phosphatase, *E2F1* E2F transcription factor 1, *EGFR* epidermal growth factor receptor, *EMT* epithelial-mesenchymal transition, *ERβ* estrogen receptor β, *EZH2* enhancer of zeste homolog 2, *GLTP* glycolipid transfer protein, *HIF-1α* hypoxia inducible factor 1 subunit alpha, *IGF* insulin-like growth factor, *JNK* c-Jun N-terminal kinases, *MAPK1* mitogen-activated protein kinase 1, *MECP2* methyl-CPG binding protein 2, *MET* MET proto-oncogene, *MLKL* mixed lineage kinase domain like pseudokinase, *NRF2* nuclear factor erythroid 2-related factor 2, *p27kip1* cyclin-dependent kinase inhibitor 1b, *PI3K* phosphatidylinositol-4,5-bisphosphate 3-kinase, *PINK1* PTEN induced kinase 1, *PRKN* Parkin, *PTEN* phosphatase and tensin homolog, *PUMA* P53 up-regulated modulator of apoptosis; *RASSF1* ras association domain family member 1, *SNAIL* snail family transcriptional repressor, *STAT* signal transducer and activator of transcription, *TWIST* twist family BHLH transcription factor, *YBX1* Y-Box binding protein1, *YTHDF2* YTH N6-methyladenosine RNA binding protein 2, *ZEB* zinc finger e-box binding homeobox.

The relationship between miRNAs dysregulation and TKI resistance is continuously being revealed. miRNAs participate in various pathological processes and are potential markers for prognosis and systemic treatment response.^6,380^miRNAs are associated with EMT. ZEB1/2 are repressors of miR-200. miR-200 targets ZEB family members by establishing double negative regulatory feedback loops to confine ZEB expression. A similar scenario exists between TWIST and miR-186, SNAIL and miR-34a. On the contrary, three EMT-TF/miRNA groups form a self-enforcing regulatory network.^247^ SH3 domain-containing kinase binding protein 1 (SHKBP1) is a direct target of miR-499a that ZEB1 inhibits. SHKBP1 regulates EGFR activity. TGF-β upregulates ZEB1 in osteosarcoma, which leads to higher expression of SHKBP1 through miR-499a. SHKBP1 also increases EGFR activity, which occurs concomitantly with a TGF-β-induced EMT-associated kinase switch to an AKT-activated with EGFR-independent state. Meanwhile, AKT upregulates SHKBP1 expression, enhancing EGFR activity.^381^ In HCC, upregulation of miRNA-125-5p suppressed ataxin-1 expression and induced Snail-mediated EMT, enhancing cancer cell stemness and leading to sorafenib resistance.^382^ In addition, miR-424 could target chromo box 4 (CBX4) to induce YAP nuclear translocation, which enhances tumor stemness and sorafenib resistance.^383^ Additionally, tumor cells can release miRNAs from exosome to TME and convey resistance to other cells. For example, cancer cells-released miR-25-3p and miR-130b-3p can promote TAM-M2 polarization, enhance VEGF expression, and induce EMT, thus leading to multidrug resistance in colon cancer.^384^

miRNA could regulate cell death. miR-125d-5p, miR-494, and miR-30a-5p overexpression in HCC cell lines are associated with sorafenib resistance. Among them, miR-125d-5p inhibits c-Jun-mediated PUMA transcriptional induction. Low levels of PUMA increase mitochondrial membrane potential and decrease ROS levels, leading to apoptosis resistance.^385^ miR-494 inhibits PTEN expression, thus activating PI3K/Akt/mTOR pathway, and downregulates PUMA to resist apoptosis.^386^ miR-30a-5p downregulation leads to high CLCF1 expression, which activates GLUT3 and HK2 expression through the PI3K/AKT pathway to enhance glycolysis-induced drug resistance.^387^ Besides, miR-30c is highly expressed in NSCLC with MET mutations, and it directly targets the 3’-untranslated region of BIM to inhibit its transcription, leading to apoptosis resistance.^388^ Moreover, miR-335 regulates AXL expression, which leads to EGFR-TKI resistance in NSCLC.^389^ In breast cancer cell lines, lapatinib induces Src to activate the NF-κB pathway to upregulate miR-221. High miR-221 leads to death resistance by inhibiting cyclin-dependent kinase inhibitor 1B (p27^kip1^).^390^ Additionally, studies have shown that activation of the NRF2 pathway, which is related to ferroptosis, increased miR-196a expression. Glycolipid transfer protein (GLTP) was a direct target of miR-196a, whose downregulation led to NSCLC resistance to gefitinib.^391^ The detail of GLTP in TKI resistance hasn’t been clarified, but it is involved in several processes (autophagy, inflammation, cell death, etc.) and may be a drug target.^392^

Apart from miRNAs, other ncRNAs like lncRNAs and circRNAs are associated with TKI-related drug resistance.^393^ In sunitinib-treated RCC, lncRNA-ECVSR can enhance the stability of estrogen receptor beta (Erβ) mRNA to upregulate HIF-2α transcription, promote vascular mimicry, cancer stemness, and RCC progression.^394^ LncRNA-CASC9 suppresses DUSP1 expression by recruiting histone methyltransferase enhancer of zeste homolog 2 (EZH2). Decreased DUSP1 expression results in continuous RAS/MAPK activation and causes gefitinib resistance in NSCLC.^395^ Moreover, lncRNA-PCAT-1 may increase the phosphorylation of AKT and GSK3, resulting in sustained activation of PI3K/AKT and gefitinib resistance.^396^ LncRNA-HIF1A-AS2 induces IL-6 expression by binding to miR-146b-4p and activating the IL-6/STAT pathway. IL-6 overexpression results in LUAD resistance.^397^ High lncRNA-RP11616M22.7 are associated with imatinib resistance in GIST, which may lie in the regulation of the hippo pathway by Ras association domain family member 1 (RASSF1).^398^ As for circRNA, sorafenib induces circFN1 and circFOXM1 expression in HCC, thereby promoting E2F transcription factor 1 (E2F1) and methyl-CpG binding protein 2 (MECP2) expression through circFN1/miR-1205/E2F1 and circFOXM1/miR-1324/MECP2 pathways. These molecular interactions were shown to promote cell proliferation, metastasis, and apoptosis resistance.^399,400^ CircRNA hsa_circ_0005576 directly interacts with miR-512-5p in LUAD cell lines and upregulates miR-512-5p/IGF-1R pathway, leading to osimertinib resistance.^401^ In addition, circRNA-SORE binds to Y-box binding protein 1 (YBX1) to block the interaction between YBX1 and the pre-mRNA-processing factor 19 (PRP19) (an E3 ubiquitin ligase).^402^ Consequently, PRP19-mediated YBX degradation is inhibited, and high YBX is related to the apoptosis resistance.^403^ Moreover, circRNA-SORE has also been shown to convey sorafenib resistance to other HCC cells via exosomes.^402^ In addition to its posttranscriptional regulatory function, circRNA can directly encode proteins.^404^ The secreted E-calmodulin variant protein (C-E-Cad) encoded by circular E-cadherin (circ-E-Cad) RNA was found to bind to the EGFR CR2 structural domain to activate EGFR without EGF, provoking GBM resistance to EGFR therapy.^405,406^

By sharing common signaling molecules, different ncRNA regulatory axes form an extensive ncRNA regulatory network that regulates TKI resistance. Preliminary circRNA/lncRNA-miRNA–mRNA networks have been constructed to shed light on the role of ncRNAs in regulating signaling pathways and drug resistance.^407^

Given the complicated and delicate regulatory mechanisms of ncRNAs and their involvement in regulating multiple pathways, ncRNAs are promising therapeutic targets. Some ncRNA drugs have been used in clinical trials but not for treating TKI-resistant tumors. Preclinical studies have shown the therapeutic potential of ncRNAs. For example, increased miR-424 levels induced CBX4 depolymerization to reduce YAP1 nuclear translocation, and improved sorafenib resistance.^383^ MiR-210-5p antagomir in combination with sorafenib alleviated sorafenib resistance.^347^ In addition, paeonol inhibits proliferation and promotes apoptosis of apatinib-resistant gastric cancer cells via lncRNA-00665/miR-665/MAPK1 axis.^408^ CircKCNN2 upregulates methyl-CpG binding domain protein 2 (MBD2) via miR-520c-3p. miR-520c-3p mediates epigenetic transcriptional silencing of FGFR4, which increases the efficacy of lenvatinib in HCC.^409^ In addition, ncRNAs have a prognostic value. Studies have shown that elevated levels of circulating miR-125d-5p often predict a low response to sorafenib in HCC, and antagonizing miR-125d-5p may attenuate drug resistance.^385^ In short, modulating ncRNA expression by ncRNA antagonists, analogs, or by targeting ncRNA upstream regulators can help reverse drug resistance.

#### Other epigenetic modifications

Other types of epigenetic modifications such as DNA methylation, histone modification, RNA methylation, and chromatin remodeling are also involved in TKI resistance.

DNA methylation is one of the first epigenetic regulatory mechanisms affecting gene transcriptional activity. Studies have shown that compared with other NSCLC cell lines with the methylated EGFR promoter region, NSCLC cell lines with the unmethylated EGFR promoter region are more sensitive to gefitinib, indicating that EGFR promoter methylation is a potential mechanism of acquired resistance.^410^ Aberrant methylation of gamma-aminobutyric acid type B receptor subunit 2 (GABBR2) can be seen in erlotinib-treated NSCLC cell lines, which leads to its overexpression. High GABBR2 activates ERK phosphorylation, significantly preventing erlotinib-induced apoptosis.^411^ Moreover, in HCC, hypermethylation of the CPG island at the ADAMTS-like 5 (ADAMTSL5) locus results in its overexpression, but reduced expression and/or phosphorylation of several RTKs (MET, EGFR, PDGFRβ, IGF1Rβ, and FGFR4) and increased sensitivity to sorafenib, lenvatinib, and regorafenib were witnessed when low the ADAMTSL5 level,^412^ suggesting that ADAMTSL5 may contribute to TKI resistance through regulating several RTKs activity.

Histone modifications regulate gene transcription by altering chromatin structure. Abnormal histone modifications are associated with TKI resistance. Histone methylation mediated by histone methyltransferase (HMT) may contribute to TKI resistance. Set domain containing 2 (SETD2), an HMT responsible for trimethylation modification of lysine at position 20 of histone H3 (H3K20me3), leads to transcriptional repression. Low SETD2 promotes the MYCN (n-myc) transcription in CML cells. MYCN can promote proliferation, immortalization, stemness, and carcinogenesis and aggravate imatinib resistance.^413^ However, the effect of MYCN upregulation on CML resistance has not been fully elucidated. Furthermore, the EZH2 is an HMT responsible for trimethylation modification of lysine at position 27 of histone H3 (H3K27me3). In HCC, HOXB cluster antisense RNA 3 (HOXB-AS3) interacts with EZH2 to recruit EZH2 to the Dicer’s promoter to add H3K27me3 modification, leading to Dicer transcriptional repression, resulting in the acquisition of stem cell-like properties and sorafenib resistance.^414^ Dicer is an RNase III nucleic acid endonuclease in the cytoplasm that is essential for mRNA maturation. However, there are still gaps in the relationship between Dicer downregulation and TKI resistance. Additionally, deletion of lysine methyltransferase 5 C (KMT5C), an HMT responsible for histone H4 lysine 20 trimethylation (H4K20me3), upregulates lncRNA-LINC01510 and promotes MET transcription, leading to EGFR-TKI resistance in NSCLC through activation of the bypass pathway.^415^ SET and MYND domain containing 2 (SMYD2) are highly expressed in ccRCC, and associated with multidrug resistance, including sunitinib in RCC. Mechanistically, SMYD2 binds to the promoter of miR-125b, inhibiting the expression of Dickkopf WNT signaling pathway inhibitor 3 (DKK3) and promoting proliferation and migration.^416^

Notably, a vitro experiment revealed changes in epigenetic modifications of GB with ALK kinase mutations and MYCN amplification from drug sensitivity to drug resistance under intervention with an ALK inhibitor. MYCN expression was initially reduced due to H3K27me3 of its locus, and gradually, the promoter of the brother of the regulator of the imprinted site (BORIS) was hypermethylated and further promoted BORIS expression. BORIS could bind to chromatin loop anchors of the DNA regulatory region containing super-enhancers and promote the expression of genes in this region, resulting in full drug resistance.^417,418^ This study showed progressive epigenetic modifications in tumors from drug sensitivity to complete drug resistance, supporting the role of epigenetic modifications in reversible drug resistance.

Abnormal histone acetylation modifications also lead to TKI resistance. Studies have shown that high CD13 is associated with sorafenib resistance in HCC. Mechanistically, CD13 stabilizes histone deacetylase 5 (HDAC5), which is critical in regulating LSD1 protein stability through post-translational modification. LSD demethylates the NF-κB catalytic unit p65 to activate the NF-κB pathway in an HDAC5-dependent manner, resulting in cell survival.^419^ In addition, upregulated lysine demethylase 6 A (KDM6A) in CML can be recruited to the NTRK1 promoter by the transcription factor YY1. KDM6A enhances NTRK1 function and induces imatinib resistance by bypassing pathways.^420^

RNA modification is a vital process that regulates posttranscriptional gene expression. mRNA N6-methyladenosine (m6A) modification can affect gene transcription by influencing mRNA stability, and m6A methyltransferase (METTL) plays an important role in TKI resistance. METTL3 is overexpressed in NSCLC, and it positively regulates autophagy by strengthening the stability of autophagy-related mRNAs such as ATG5 and ATG7, resulting in gefitinib resistance.^421^ METTL3 also changes MET levels to modulate PI3K/AKT pathway, leading to LUAD resistance to gefitinib.^422^ In addition, METTL3 upregulates AXL expression and induces EMT in ovarian cancer.^423^ Similarly, METTL7B can increase the expression of GPX4, HMOX1, and SOD1 by m6A modification, leading to ferroptosis resistance and TKI resistance in LUAD (see Ferroptosis).^424^ Moreover, METTL-KIAA1429 can enhance homeobox A1 (HOXA1) mRNA stability and lead to gefitinib resistance in NSCLC through HOXA1 expression,^425^ but HOXA1 is considered an oncogene whose function is currently unknown. The m6A modification mediated by YTH N6-methyladenosine RNA binding protein 2 (YTHDF2) can increase circRNA-ASK1 degradation, thereby attenuating the inhibitory effect of the circRNA-ASK1-encoded product ASK1-272a.a on AKT pathway and increasing LUAD resistance to gefitinib.^426^ YTHDF also mediates m6A modification of lncRNA-TUSC7 and abolishes the inhibitory effect of miRNA-146 on the Notch pathway, whose activation is related to EMT and LUAD resistance to erlotinib.^427^

FTO alpha-ketoglutarate-dependent dioxygenase (FTO) is an m6A demethylase, and it can be induced to express by TKIs in leukemia. FTO increases the stability of cell survival- and proliferation-related mRNAs through m6A demethylation, allowing cells to rapidly upregulate the expression of anti-apoptotic molecules such as BCL-2 and proliferative genes such as MERTK. This enables cells to withstand the initial TKI attack. Moreover, anti-apoptotic gene expression can be maintained, developing a reversible drug-resistant phenotype. An FTO inhibitor, rehein, can restore TKI sensitivity.^428^ Moreover, FTO can enhance FLT3-ITD expression to promote acute promyelocytic leukemia (APL) cell survival and proliferation. FTO-mediated post-transcriptional repression of retinoic acid receptor alpha (RARA), ankyrin repeat and SOCS box containing 2 (ASB2) are involved in the upregulation of FLT3-ITD.^429^ As an m6A reader, insulin-like growth factor binding protein 2 (IGFBP2) enhances ERBB2 mRNA translation, induces HER2 expression, and leads to selumetinib resistance. Lapatinib targets both IGFBP2 and BRBB2 to reverse selumetinib resistance.^430^

RNA binding protein (RBP) is also a kind of RNA modification. RBP MUSASHI-2 (MSI2) binds to the mRNA of Nanog (Nanog homeobox), a core stem cell factor. High Nanog is associated with cancer stem cell-like properties and leads to death resistance, resulting in osimertinib resistance of LUAD, and drug sensitivity restored after MSI knockdown.^431^

Chromatin remodeling in GB may be associated with reversible chronic tumor resistance to TKI. Under the TKI therapy, some GB cells gradually transform into reversibly resistant cells with upregulation of histone demethylase (KDM), extensive redistribution of H3K27me3, and overactivation of the Notch pathway. KDM and H3K27me3 increase target gene expression. The notch pathway may be associated with stem cell properties acquirement.^432^ The link between these alterations and drug resistance needs further investigation.

So far, some epigenetic drugs have been approved for cancer treatment, and some preclinical studies showed the promising efficacy of combination therapy with TKIs and epigenetic drugs. Aberrant DNA hypermethylation is associated with lower TKI sensitivity, and imatinib combined with the DNA methyltransferase inhibitor (DNMTi) decitabine enhanced TKI-induced cell apoptosis and induced oncogene expression, exhibiting promising antitumor effects.^433^ Moreover, DI-1 enhanced the cabozantinib sensitivity in osteosarcoma by inhibiting DNMT-1.^434^ Intervention with histone modifications is also a potential option. EZH2 overexpression is associated with poor prognosis, providing a reason for EZH2 inhibition. In sorafenib-resistant TC cell lines, sorafenib sensitivity was restored when combined with EZH2 inhibitor.^435^ Furthermore, sorafenib has been shown to induce apoptosis in HCC cells by accelerating the EZH2 degradation, suggesting that EZH2 inhibitors in combination with TKI reverses drug resistance and potentially enhances the drug efficacy before resistance.^436^ 2,4-pyrimidinediamine derivative 10 f is a dual ALK and HDAC inhibitor. It can treat ceritinib- or crizotinib-resistant NSCLC cell lines.^437^ Tazemetostat is the first FDA-approved EZH2 inhibitor recently,^438^ whose combination with TKIs deserves further study.

Histone deacetylase inhibitors (HDACi) are suitable candidates for combination therapy. HDACis combined with EGFR-TKIs effectively overcomes TKIs resistance.^439–441^ For example, the combination of vorinostat and osimertinib attenuates apoptosis resistance in NSCLC cell lines with BIM deletion polymorphisms.^442^ The combination of vorinostat and brigatinib has been effective in LUAD cell lines with third-generation EGFR-TKIs resistance mutations.^443^ Epigenetic regulatory interventions are diverse, but it is clear that epigenetic manipulation is an effective strategy to restore TKI sensitivity.^444,445^

Overall, Tumor cells can acquire drug resistance through epigenetic reprogramming. However, the presence of various epigenetic modification methods makes research challenging. More importantly, epigenetic modification does not change the gene sequence. In other words, epigenetic modification is reversible, so proper epigenetic intervention may be the key to reversing drug resistance (Table 4).

**Table 4:** Preclinical studies of TKI in combination with other drugs to reverse drug resistance

TKI	Cancer	Target	Anticancer therapies	Type	anti-resistance mechanism	Ref.
Reversing EMT						
Erlotinib	NSCLC	TGF-βR	LY364947	TGF-β inhibitor	Inhibiting TGF-β and reversing EMT	^268^
Selumetinib	NSCLC	AXL	bemcentinib	AXL TKI	Reducing TGF-β and blockade	^271^
EGFR-TKI	NSCLC	AuroraA	alisertib	AuroraA kinase inhibitor	reversing EMT	^272^
Osimertinib	NSCLC	AuroraB	GSK1070916 ENMD-2076 PF-003814735	AuroraB kinase inhibitor	reversing EMT	^253^
Crizotinib Alectinib Ceritinib	NSCLC	AXL	Ganetespib R428	HSP90α inhibitor AXL-TKI	Inhibiting AXL and reversing EMT	^266^
Sorafenib	HCC	AXL	R428	AXL-TKI	Inhibiting AXL and reversing EMT	^267^
Erlotinib	HNSCC	TrkA	siTrkA1, siTrkA3	TrkA siRNA	Reversing EMT through blockading NGF/TrkA/STAT3 pathway	^170^
sunitinib	RCC	Plk1	ab19777 NB100-122	Plk1 antibody HIF-2α antibody	Reversing EMT through blockading HIF-2α/ Plk1	^257^
Metabolism intervention						
regorafenib	HCC	HK1	HK1 shRNA	HK1 shRNA	Intervening metabolism to reverse drug resistance	^277^
Imatinib	CML	PKM2	PKM2 siRNA	PKM2 siRNA	Reducing glycolysis and PI3K / AKT pathway activity and inducing apoptosis	^279^
Gefitinib	NSCLC	lactate	NHI-Glc-2	LDH inhibitor	Reducing the secretion of HGF of CAFs by reducing the stimulation of lactate	^275^
Gefitinib Erlotinib	NSCLC	OXPHOS complex I	Phenformin	OXPHOS inhibitor	Inhibiting proliferation by inhibiting mitochondrial OXPHOS	^284^
Gefitinib	NSCLC	SREBP	Anti-SREBP1	SREBP1antibody	Intervening lipid metabolism to induce ROS production and induce apoptosis	^289^
Gefitinib	NSCLC	PPARα	fenofibrate	lipid-lowering drug	Intervening cholesterol metabolism and induces apoptosis	^291^
Blocking aberrant activation of downstream pathways						
Imatinib	CML	mTOR	everolimus	mTOR inhibitor	Inducing apoptosis and cell cycle arrest by blockading mTOR	^106^
Sunitinib	RCC	mTOR	Rapalink-1	mTOR inhibitor	Inducing apoptosis by blockading mTOR, MAPK signaling pathway	^109^
Gefitinib Osimertinib	NSCLC	FGFR, AKT	PD173074 GSK2141795	FGFR TKI AKT inhibitor	Blockading PI3K/AKT signaling pathway through dual inhibition of FGFR and AKT	^110^
Sorafenib	HCC	MET, MET	MK2206 capmatinib	AKT inhibitor MET TKI	Blocking bypass activation of downstream signaling pathway	^111^
BCR-ABL TKI	AML	RAF	asciminib	RAF inhibitor	Blockading RAS/MAPK signaling pathway through inhibition of RAF	^113^
Regorafenib	gastric cancerr	MEK	Erdafitinib Trametinib	FGFR TKI MEK inhibitor	Blockading RAS/MAPK signaling pathway through dual inhibition of FGFR and MEK	^116^
erlotinib	ATC	MEK	Trametinib	MEK inhibitor	Blockading RAS/MAPK signaling pathway through dual inhibition of EGFR and MEK	^117^
entrectinib	PDAC	MEK	cobimetinib	MEK inhibitor	Blockading RAS/MAPK signaling pathway through dual inhibition of NTRK1 and MEK	^118^
Dasatinib	EC	MEK	Trametinib	MEK inhibitor	Blockading RAS/MAPK signaling pathway through dual inhibition of EphA2 and MEK	^119^
Dasatinib	NB	ERK	ulixertinib	ERK inhibitor	Blockading MEK/ERK signaling pathway which activated by EPO/NGF through inhibition of ERK	^114^
Lenvatinib	ATC	PAK	KPT-9274	PAK inhibitor	Blockading RAS signaling pathway and promote apoptosis	^122^
EGFR-TKI	NSCLC	MEK, PI3K	MEK162 BKM120	MEK inhibitor PI3K inhibitor	Dual inhibition of PI3K/AKT and RAS/MAPK pathways	^123^
Lapatinib	GIST	MEK, PI3K	Refametinib copanlisib	MEK inhibitor PI3K inhibitor	Dual inhibition of PI3K/AKT and RAS/MAPK pathways	^124^
ALK-TKI	ALCL	SHP3	SHP3 inhibitor	SHP3 inhibitor	Inhibiting the RAS pathway overactivation	^136^
ALK-TKI	NB	PIM	AZD1208	PIM inhibitor	Blockading JAK/STAT3 signaling pathway	^171^
Gefitinib	GB	IGFIRβ	Anti-IGFIRβ	IGFIRβ antibody	Enhancing the inhibition of AKT and MEK/ERK pathway, inducing apoptosis	^183^
Lenvatinib	HCC	EGFR	Gefitinib	EGFR-TKI	Blocking the bypass activation of ERK pathway by inhibiting the EGFR	^186^
gefitinib		FAK	defactinib	FAK-TKI	Enhancing the inhibition of AKT and MEK/ERK pathway, inducing apoptosis	
regorafenib	CRC	IGFR	aspirin	IGF inhibitor	Enhancing the inhibition of AKT and MEK/ERK pathway, inducing apoptosis	^189^
Osimertinib	NSCLC	MET	berberine	MET inhibitor	Blockading the bypass activation caused by MET-amplification	^190^
imatinib	GIST	HSP90	TAS-116	HSP90 inhibitor	Blockading the bypass activation caused by aberrant activation of KIT overexpression	^191^
Lapatinib	Brest cancer	HSP90	17-DMAG	HSP90 inhibitor	Not clear, inhibiting the cell proliferation and protein expression	^192^
Alectinib	NSCLC	GAB	Phenformin	GAB inhibitor	Blockading the bypass activation caused by aberrant activation of HGF/MET	^193^
Induction of cell death						
EGFR-TKI	NSCLC	BIM BCL-2	Vorinostat Metformin	Bim inducer Bcl-2 inhibitor	Inducing apoptosis	^308^
alectinib	NSCLC	NOXA	ixazomib	proteasome inhibitor	Inducing apoptosis by increasing the expression of NOXA	^310^
regofenib	CRC	MCL-1	S63845	MCL-1 inhibitor	Inducing apoptosis by decreasing the expression of MCL-1	^311^
Sorafenib	HCC	Mitochondria	piperlongumine	–	Inducing ROS generation, mitochondrial dysfunction and cell cycle arrest, activating the AMPK signaling pathway.	^313^
crizotinib	LUAD	CDK	THZ1 Alvocidib dinaciclib	CDK7/12 inhibitor CDK9 inhibitor	Inducing apoptosis by cell cycle arrest	^314^
lapatinib	Breast cancer	CDK	THZ1	CDK7 inhibitor	Inducing apoptosis by cell cycle arrest	^315^
lapatinib	Breast cancer	CDK	dinaciclib	CDK12 inhibitor	Enhancing inhibition of PI3K/AKT pathway and inducing apoptosis	^316^
Gefitinib	NSCLC	GPX4	fluvastatin	GPX4 inhibitor	Inducing ferroptosis	^331^
lapatinib	Breast cancer	Transferrin ferroportin-1	Siramesine	lysosome disrupting agent	Increasing the intake of Fe and inducing ferroptosis	^335^
sorafenib	HCC	Mitochondria ML385	DSF/Cu	Mitochondrial functional interference agents NRF inhibitor	Increasing ROS generation, mitochondrial dysfunction and inhibiting antioxidant effect of NRF pathway to induce ferroptosis	^336^
Gefitinib	NSCLC	Muti-target	CTS	–	Decreasing the expression of CAT, HMOX1 and SCD to enhance vulnerability to oxidative stress	^294^
Sorafenib	HCC	–	amiloride	Micropinocytosis inhibitor	Inhibiting micropinocytosis to reducing the intracellular cysteine intake and inducing ferroptosis	^330^
afatinib	LUAD	–	CQ	Autophagy inhibitor	Inhibiting autophagy to induce cell death	^341^
gefitinib	Pancreatic cancer	–	azithromycin	Autophagy inhibitor	Inhibiting autophagy to induce cell death	^348^
imatinib	CML		cannabidiol	Autophagy inducer	Inducing autophagy to induce cell death	^354^
quizartinib	CML	–	bortezomib	proteasome inhibitor	Inducing autophagy to enhance degradation of FLT3-ITD	^355^
sorafenib	HCC	HSP90α	17-AAG	HSP90α inhibitor	Inducing necroptosis	
Sunitinib	RCC	–	Shikonin	-–	Inducing necroptosis and inhibiting mTOR	^367^
Erlotinib	NSCLC	APE	NO.0449-0145	APE inhibitor	Inducing necroptosis, apoptosis, pyroptosis	^368^
Intervention of epigenetic modifications						
Imatinib	CML	DNMT	decitabine	DNMTis	Inducing apoptosis and expression of tumor suppressor gene	^433^
cabozatinib	OS	DNMT-1	DI-1	DNMTis	Enhancing sensitivity to TKI	^434^
Sorafenib	TC	EZH2	EPZ-6438	EZH2 inhibitor	decreasing H3K27me3 and increasing H3K27ac levels	^435^
Osimertinib	NSCLC	HDAC	SAHA	HDACi	Inhibiting histone deacetylase and autophagy	^439^
Gefitinib	NSCLC	HDAC	GCJ-490A	HDACi	Increasing histone acetylation at the IKKα promoter and enhancing IKKα transcription, through which MET level decreased	^440^
Osimertinib	LUAD	HDAC3	vorinostat	HDACi	Increasing BIM expression and inducing apoptosis	^443^
EGFR-TKI	LUAD	HDAC ALK	Vorinostat brigatinib	HDACi ALK-TKI	Inhibiting PI3K/AKT pathway and inducing apoptosis	^443^

*17-AAG* 17-demethoxygeldanamycin, *AKT* AKT serine/threonine kinase, *ALCL* anaplastic large cell lymphoma, *ALK* anaplastic lymphoma receptor tyrosine kinase, *APE1* apurinic/apyrimidinic endonuclease 1, *ATC* anaplastic thyroid carcinoma, *AXL* AXL receptor tyrosine kinase, *BIM* bcl-2 interacting mediator of cell death, *CAF* cancer-associated fibroblasts, *CAT* catalase, *CDK* cyclin-dependent kinase, *CML* chronic myelocytic leukemia, *CQ* chloroquine, *CRC* colorectal cancer, *DNMTi* DNA methyltransferase inhibitors, *EC* endometrial cancer, *EMT* epithelial-mesenchymal transition, *EPO* erythropoietin, *EZH2* enhancer of zeste homolog 2, *FAK* focal adhesion kinas, *FGFR* fibroblast growth factor receptor, *FGFR* fibroblast growth factor receptor, *GAB1* GEB2 associated binding protein 1, *GBM* glioblastoma, *GPX4* glutathione peroxidase 4, *H3K27me3* trimethylation of lys-27 in histone 3, *HCC* hepatocellular carcinoma, *HDACi* histone deacetylase inhibitors, *HGF* hepatocyte growth factor, *HK1* hexokinase 1, *HMOX1* heme oxygenase1, *HSP70* heat shock protein 70, *IGFR* insulin-like gOriginal rowth factor receptor, *KIT* KIT proto-oncogene; *LDH* lactate dehydrogenase, *LUAD* lung adenocarcinoma, *MCL-1* mcl1 apoptosis regulator, *MEK* MAP kinase kinase, *MET* MET proto-oncogene, *mTOR* mechanistic target of rapamycin kinase, *NB* neuroblastoma, *NGF* nerve growth factor, *NOXA* NADPH oxidase activator, NSCLC non-small cell lung cancer, *NTRK*:neurotrophic receptor tyrosine kinase 1, *OS* osteosarcoma, *OXPHOS* mitochondrial oxidative phosphorylation system, *PAK1* P21 (RAC1) activated kinase 1,*PDAC* pancreatic ductal adenocarcinoma, *PIM* Pim-1 proto-oncogene, *PKM2* pyruvate kinase m2, *Plk1* polo-like kinase 1, *PPARα* peroxisome proliferator activated receptor alpha, *RCC* renal cell carcinoma, *ROS* reactive oxygen species, *SCD1* stearoyl-coA desaturase, *SREBP1* sterol regulatory element binding protein 1, *TC* thyroid cancer, *TGF-β* transforming growth factor beta, *TrkA* tropomyosin receptor kinase A.

### Abnormal metabolism of TKIs

#### Drug delivery

Absorption, distribution, metabolism, and excretion (ADME) of TKIs require trans-membrane transport through transporter proteins, and transporter proteins can also exacerbate drug resistance by impeding trans-membrane transport of drugs. Drug transporters located in enterocytes, hepatocytes, renal proximal tubular cells, and physiological barriers such as the blood-brain barrier are directly involved in drug absorption, distribution, and elimination. They also regulate the intracellular concentration of metabolic enzymes, thereby indirectly affecting drug metabolism.^8^ The altered activity of transporter proteins can decrease the absorption of TKIs or increase their excretion. Thus, TKI concentrations in circulation and targeted regions are inadequate to inhibit abnormal PTKs completely. Furthermore, drug transporters are essential for TKI uptake or excretion by cancer cells. Reduced influx or increased efflux of TKIs mediated by drug transporters in cancer cell membranes can lead to drug resistance. Various mechanisms, such as genetic variations, epigenetic factors, and transporter-mediated drug interactions, can affect the expression and function of drug transporters in cancer cells, resulting in different clinical outcomes.^8^

Theoretically, reducing the activity or expression of transporter proteins can decrease intracellular drug accumulation.^443^ Several studies have shown that the solute carrier (SLC) transporter proteins are responsible for TKI uptake by cancer cells,^8^ and weak clinical response of CML to imatinib was associated with reduced SLC22A1 expression and activity.^446^ However, most TKIs cannot be considered the substrates of SLC, because TKIs concentration decreased in most cells transfected with SLC.^8^ Therefore, further studies are needed to clarify the cellular mechanisms of TKI uptake and to measure the effect of transporter proteins on TKI resistance.

Compared with drug influx, drug efflux is considered a more prevalent sector of drug resistance. Increased drug efflux can reduce its intracellular concentrations below the therapeutic levels, resulting in drug resistance. The efflux of TKIs mainly relies on the ATP binding cassette (ABC) family proteins, such as ATP binding cassette subfamily B member 1 (ABCB1, p-gp) and breast cancer resistance protein (BCRP).^447^ Single nucleotide polymorphisms (SNPs) of ABC may affect TKI efflux. For instance, 1199 G > A SNP in ABCB1 increased imatinib, nilotinib, and dasatinib efflux compared with the wild genotype.^448^ However, 421 G > A SNP in ABC subfamily G member 2 (ABCG2) reduced the affinity of TKIs to ABCG2 and prevented drug resistance compared with wild genotype.^449^ It may be because SNP in the ATPase structural domain of ABCG2 can affect ABCG activity to reduce drug efflux.^450^ In addition, increased expression or enhanced function of p-gp can also facilitate drug efflux and drug resistance.^441,442^ Gene rearrangements can lead to the insertion of a more active promoter in MDR-1 (p-gp) mRNA,^451,452^ which can still translate biologically active abnormal p-gp to increase drug efflux.^453^ It may be a reason for ceritinib resistance in NSCLC cell lines with ALK rearrangement.^454^ Epigenetic modifications also regulate ABC expression. MiR-212 is downregulated in imatinib-treated CML, which could upregulate ABCG2 and lead to resistance.^455^ Many factors affect the function of drug transporters, but the relationship between some of them and TKI resistance needs further research. Interestingly, research in sunitinib-resistant metastatic RCC cell lines indicated a novel way of TKIs efflux. Sunitinib promotes TFE3 (transcription factor binding to IGHM enhancer 3) translocation into the nucleus, increasing the expression of extended-synaptotagmin-1 (ESYT1) that is an endoplasmic reticulum protein. ESTY1 formed a dimer with synaptotagmin-7 (SYT7) on the lysosome and induced endoplasmic reticulum breakage, Ca^2+^ release, and lysosomal exocytosis. Lysosomal exocytosis can increase sunitinib efflux and simultaneously release histone B out of the cell to degrade the ECM and facilitate tumor metastasis, and Ca^2+^ induces lysosomal biogenesis and forms a positive feedback.^456^

Pregnane X receptor (PXR), a transcriptional activator, can sense and remove foreign substances by regulating the expression of drug-metabolizing enzymes and drug transporters.^457^ In prostate cancer, PXR leads to dasatinib resistance but increases erlotinib, dabrafenib, and afatinib sensitivity. PXR-induced upregulation of cytochrome p450 family three subfamilies A member 4 (CYP3A4) may lead to dasatinib resistance. CYP3A4 metabolizes dasatinib to its inactive form, and PXR increases dasatinib efflux. In contrast, afatinib sensitivity may be because of PXR-induced upregulation of SLC16A1, which increases the intracellular concentration of afatinib. PXR also increases the active form of afatinib in a CYP-dependent manner.^458^ It supports the concept that drugs can enter tumor cells differently, and the pharmacokinetic parameters of different TKIs can affect their clinical efficacy.

Despite the role of ABC inhibition in reversing drug resistance, ABC inhibitors almost failed in clinical trials because of their serious adverse effects and drug interactions.^459^ ABC transporters are not specifically expressed on tumor cells, but they are also widely distributed in other cells such as hepatic and intestinal cells, which are related to drug uptake and efflux. TKI delivery by nanocarriers may improve the situation. Nanocarriers enter the cells by endocytosis, which simultaneously maintains targeting and attenuates the influence of transporter proteins.^460^ In addition, some TKIs rely on ABC to out the cell but inhibit ABC at the same time. Studies have shown that nilotinib is a potent inhibitor of P-gp and BCRP, and concurrent administration of nilotinib with afatinib in animal models reduced the clearance of afatinib by 65.3%.^461^ The possible reason lies in the homology of the ATP binding sites of ABC and TKI-targeted PTKs, implicating that combinations or higher doses of TKIs may be effective.^462^ However, most of these studies were conducted in vitro and could not measure the impact of ABCs on drug uptake and efflux in different organs. Therefore, in vivo studies are needed to clarify the relationship between TKI and ABC.

#### Lysosomal sequestration

Lysosomes are acidic digestive vesicles composed of lipoprotein membranes and over 50 hydrolytic enzymes. Hydrophobic, weakly basic TKIs can enter the lysosomes by pH gradient-driven free diffusion or ABC transporters. Thereafter, protonated TKIs lose their ability to penetrate the membrane, being isolated in vesicles far from their target, named lysosomal sequestration. Several TKIs such as sunitinib, erlotinib, and sorafenib undergo lysosomal sequestration.^463^ In addition, protonated TKIs in the lysosomes can prevent the mTOR complex (mTORc)-mediated inhibition of transcription factor EB (TFEB). TFEB is responsible for lysosomal biogenesis. It can also increase lysosomal membrane permeability, leading to calcium expelling to activate calcineurin (CaN). Activated CaN dephosphorylates TFEB.^464^ Dephosphorylated TFEB enters the nucleus and stimulates lysosomal biogenesis, sequesters more TKIs, and exacerbates drug resistance. But such resistance is reversible. Drug-free cultures of sunitinib-resistant tumor cells can normalize the lysosomal capacity and restore drug sensitivity upon redosing.^463^

**Fig. 6:**
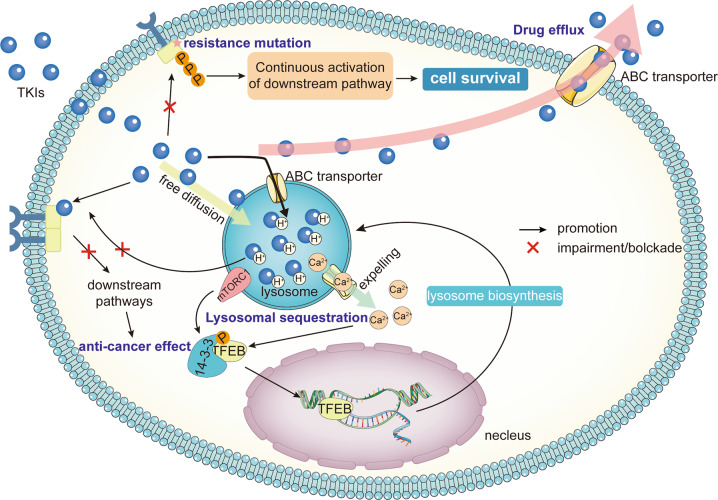
Abnormal TKI drug metabolism. After entering the cell, TKI needs to compete with ATP to bind to the ATP site of abnormal PTK to block the downstream pathway. When the TKI binding site is mutated, it will result in the inability of the TKI to bind and drug resistance will develop. In addition, after TKI enters the cell, the weakly basic TKI will be driven into the acidic lysosomes and protonated and lose the ability to cross the membrane, which leads to a decrease in intracellular TKI on the one hand and stimulates lysosome production on the other, forming a vicious circle. In addition, tumor cells can also promote TKI efflux from cells by high expression of drug efflux proteins, such as ABC, further reducing TKI concentration

Inhibition of lysosomal sequestration is an effective strategy to attenuate drug resistance. The combination of nintedanib and inhibitors of lysosomal acidification, such as bafilomycin A1 or chloroquine (CQ), significantly inhibited tumor growth in nintedanib-resistant tumors in mice.^465^ Bafilomycin A1 is an inhibitor of H^+^-ATPase in the lysosomal membrane and attenuates the acidic condition of the lysosome. Although bafilomycin A1 cannot be used for clinical purposes due to its toxicity, hydroxychloroquine (HCQ) and its derivatives have shown promising results in clinical trials.^349^ The mechanism responsible for HCQ function is unclear, but it is assumed that HCQ prevents lysosomal accumulation of the drug.^466^ Moreover, drugs targeting other components of the lysosome, such as V-ATPase, acid sphingomyelinase, cathepsin, and HSP70, markedly impair lysosomal degradation.^466^ Future studies are needed to uncover the effects of these drugs on TKI resistance. Moreover, it has been shown that different TKIs accumulate in different cellular components,^467^ and lysosomal accumulation seems to be related to the TKI’s base dissociation constant (pkb). The lysosomal accumulation of the drug increases with pkb.^462^ Therefore, assessing the physicochemical properties of TKI and modifying it may prevent lysosomal sequestration of the drug (Fig. 6).^462^

#### Drug-drug interaction (DDI)

Generally, oncology patients require several drugs to achieve a better outcome. Drugs compete with each other for protein binding, and it’s where DDI limits or enhances drug efficacy. DDI can be divided into PK-DDI and pharmacodynamics (PD) DDI.^468^ PK-DDI involves the entire process of drug ADME. PD-DDI is associated with synergistic, antagonistic, or additive reactions between drugs.^469^ DDI-mediated reduction in drug efficacy is multilayered, and some patients may not achieve optimal efficacy as a result.^470^

TKIs are mostly administered orally, and their absorption is inevitably affected by gastric acid PH, drug transporters, and intestinal enzymes.^469^ Suffering from gastrointestinal adverse effects such as gastroesophageal reflux, many patients take acid-reducing agents (ARAs) during cancer treatment. But ARA-mediated gastric acid pH increase can reduce the solubility of the weakly basic TKI and affect drug absorption and efficacy.^471^ An early study examined the effect of the omeprazole, a proton pump inhibitor (PPI), on the PK of 100 mg dasatinib in 14 healthy subjects, in which dasatinib was administered four days after the omeprazole application. A 40% decrease in the bioavailability of dasatinib was reported, and the area under the curve from zero to infinity (AUC_inf_) and peak concentration (C_max_) were reduced by 43% and 42%, respectively.^472^ Similarly, another clinical trial showed that erlotinib administered concomitantly with PPIs increased the risk of death in lung cancer patients by 21% compared with TKI alone, and discontinuation of TKI did not affect the reduced survival in this group of patients.^470,473^ That was to say, in the presence of PPI, the survival rate of lung cancer patients did not change with and without TKI use. PPI use and decreased TKI absorption may be the reason behind TKIs ineffectiveness. Moreover, the PK of erlotinib is significantly lower when administered concomitantly with PPIs and histamine H2-receptor antagonists. Therefore, concomitant use of TKIs and ARAs is not recommended in clinical.^472^ Unfortunately, there is no systematic recommendation for PPI usage during TKIs therapy, and there is no convincing prospective study measuring the interaction between PPIs and TKIs.^473^ The impact of ARAs on the efficacy of TKIs may be underestimated. Therefore, future studies focusing on the interaction between TKIs and ARAs are essential to improve the efficacy of both TKIs and ARAs.

Drug metabolizing enzymes and drug transporters are also involved in DDIs. CYP450 is the main enzyme that metabolizes TKIs. The plasma concentrations of TKIs decrease when TKIs are concomitantly administered with hepatic enzyme inducers such as rifampin, phenytoin sodium, and carbamazepine. For instance, rifampin reduces the total area under the curve (AUC) and C_max_ of bosutinib by approximately 90%.^474^ In addition, co-administration of rifampin with nintedanib reduced the AUC of nintedanib by 50% and the C_max_ by 60%, but the nintedanib degradation was lightly affected by CYP, and the phenomenon attributed to rifampicin-mediated elevated p-gp expression.^475^

In conclusion, DDI is a double-edged sword. It can enhance TKIs efficacy on the one hand, but it may reduce efficacy and enhance side effects sometimes, which lead to patients’ low compliance and treatment failure. Therefore, it is crucial to understand the pharmacokinetics of each TKI and rationally prescribe the drug in combination with other drugs.

## Outlook

TKIs are now widely used for several types of cancer. Meanwhile, TKI resistance is a major challenge in treating cancer and markedly reduces patients’ compliance, survival, and quality of life. Therefore, exploring the mechanisms behind TKI resistance is essential. Therefore, we summarize the mechanism and development of tumor with TKI resistance in this review. First, the main reason is the abnormal activation of PTK-related signaling pathways involved in gene mutations. In addition to mutations, TME is deeply involved in tumor development and TKI resistance. TME is pivotal for tumor evolution, contributes to EMT and metabolic reprogramming, and plays an important role in TKI resistance. Cell death resistance, tumor metabolism, and immune reprogramming are involved in TKI resistance and can be novel targets for drug development.^476^ Epigenetic modification is a reversible mechanism of TKI resistance that can also be a therapeutic target. Several RTKs, such as AXL and NRTKs, are primed to play an essential role in TKI resistance and are promising targets for reversing drug resistance.^477^ However, future studies are needed to provide a deeper insight into these mechanisms and their crosstalk. Besides, Appropriate therapeutic strategies can improve drug resistance. Advanced knowledge about DDI and TKIs pharmacokinetics is necessary to achieve optimal treatment efficacy. Combined therapy can also attenuate several resistance mechanisms. Herein, clinical trials using TKIs combined with other drugs or using two TKIs are ongoing. The use of TKIs with concomitant chemotherapy and immunotherapy is also of interest. However, DDI and adverse effects caused by multidrug combinations must be considered. Understanding the molecular mechanisms of the disease and relevant pharmacokinetic information is pivotal for choosing the best therapeutic approach.

Due to the heterogeneity of TKI resistance mechanisms in different types of tumors and each patient, a single therapeutic strategy cannot overcome drug resistance in all patients.^199^ Because of the individualized and dynamic nature of TKI resistance mechanisms in tumor cells and the existence of several resistance mechanisms in a single tumor cell, TKI resistance cannot be easily reversed. Precision medicine for individualized biological profiles may be helpful in the coming years. Recently, the breakthrough in biotechnology, liquid biopsies, and next-generation sequencing technologies improved the detection of TKI resistance mutations.^478^ Tumor and cell-free DNA analysis, immunolabeling, proteomics, metabolomics, and RNA analysis can identify the biological characteristics of TKI resistant tumors.^479^ They improve the identification of TKI resistance mechanisms and optimize the therapeutic approach. Based on new mechanisms of TKI resistance, better detection methods and treatment modalities are under consideration. Hence, a deeper insight into the mechanisms of TKI resistance is crucial.
